# SMOC2 promotes an epithelial-mesenchymal transition and a pro-metastatic phenotype in epithelial cells of renal cell carcinoma origin

**DOI:** 10.1038/s41419-022-05059-2

**Published:** 2022-07-22

**Authors:** Daniel Feng, Peng Gao, Nathalie Henley, Marion Dubuissez, Nan Chen, Louis-Philippe Laurin, Virginie Royal, Vincent Pichette, Casimiro Gerarduzzi

**Affiliations:** 1 Département de Pharmacologie et Physiologie, Faculté de Médecine, Université de Montréal, Montréal, Québec Canada; 2 Centre de recherche de l’Hôpital Maisonneuve-Rosemont, Faculté de Médecine, Centre affilié à l’Université de Montréal, Montréal, Québec Canada; 3 Faculty of Science, University of British Columbia, Vancouver, British Columbia Canada; 4 Département de Médecine, Faculté de Médecine, Université de Montréal, Montréal, Québec Canada

**Keywords:** Renal cell carcinoma, Metastasis

## Abstract

Renal Cell Carcinoma (RCC) is the most common form of all renal cancer cases, and well-known for its highly aggressive metastatic behavior. SMOC2 is a recently described non-structural component of the extracellular matrix (ECM) that is highly expressed during tissue remodeling processes with emerging roles in cancers, yet its role in RCC remains elusive. Using gene expression profiles from patient samples, we identified SMOC2 as being significantly expressed in RCC tissue compared to normal renal tissue, which correlated with shorter RCC patient survival. Specifically, de novo protein synthesis of SMOC2 was shown to be much higher in the tubular epithelial cells of patients with biopsy-proven RCC. More importantly, we provide evidence of SMOC2 triggering kidney epithelial cells into an epithelial-to-mesenchymal transition (EMT), a phenotype known to promote metastasis. We found that SMOC2 induced mesenchymal-like morphology and activities in both RCC and non-RCC kidney epithelial cell lines. Mechanistically, treatment of RCC cell lines ACHN and 786-O with SMOC2 (recombinant and enforced expression) caused a significant increase in EMT-markers, -matrix production, -proliferation, and -migration, which were inhibited by targeting SMOC2 by siRNA. We further characterized SMOC2 activation of EMT to occur through the integrin β3, FAK and paxillin pathway. The proliferation and metastatic potential of SMOC2 overexpressing ACHN and 786-O cell lines were validated in vivo by their significantly higher tumor growth in kidneys and systemic dissemination into other organs when compared to their respective controls. In principle, understanding the impact that SMOC2 has on EMT may lead to more evidence-based treatments and biomarkers for RCC metastasis.

## Introduction

Kidney cancer accounts for 2-3% of worldwide cancers each year of which Renal Cell Carcinoma (RCC) is the most common form, representing 90-95% of all renal cancer cases [1]. RCC is typically asymptomatic at early stages, while highly metastatic and poor prognosis for advanced RCC patients. At the time of diagnosis, 20-30% of RCC patients have metastatic disease, while another 30% with surgical extirpation will relapse and develop metastasis at follow-up [2]. RCC tumors originate primarily from the epithelial cells of the kidney, specifically the tubular cells [3]. The epithelial-mesenchymal transition (EMT) is a genetic program that promotes metastatic dissemination of malignant cells from primary epithelial tumors [4]. During EMT, cells lose their epithelial phenotype by reorganizing their adhesion and cytoskeletal structures to acquire a mesenchymal morphology and the ability to migrate. An important driver of cancer development and EMT-metastasis is the dynamic remodeling of the tumor stroma [5].

It has been increasingly accepted that the extracellular matrix (ECM) surrounding the cell is indispensable to regulating cellular behavior. Moreover, the expression profile and organization of the ECM is tightly controlled in regulating these processes during development and growth. For these reasons, it is inevitable for the ECM to be frequently deregulated and disorganized at times of cancer initiation and progression. Indeed, abnormal ECM dynamics are one of the most apparent characteristics in cancer [6]. In particular, the ECM composition and arrangement in the surrounding tumor stroma has a disturbed tissue homeostasis necessary for cancer cell motility and dissemination into secondary sites [6, 7]. In this context, inhibiting factors that regulate ECM expression and processing represents an attractive therapeutic avenue to explore against metastasis. Although significant progress has been made in understanding how growth factors and cytokines regulate the ECM, much less importance has been given to the influence matricellular proteins (MCPs) have on the matrix. MCPs are a category of ECM-associated proteins which, despite not having the classical structural role in the matrix, play an important role in regulating matrix proteins. They also modulate cell-matrix interactions to influence cellular behavior and function [8, 9]. This range of activities permits MCPs to direct a multitude of functions essential to tumorigenesis, e.g. metastasis, adhesion and migration [10], providing an explanation to the correlation between their upregulation and poor prognosis in cancer patients [11]. In fact, MCPs exhibit expression mostly in the context of tissue remodeling processes, typically induced transiently during embryogenesis and wound healing [12], but sustained in various cancers by malignant and/or tumor-associated cells [13].

Recently, an MCP called SPARC-Related Modular Calcium-Binding (SMOC2) has been identified for its emerging roles in cancers [13], yet its contribution in RCC remains elusive. SMOC2 is a member of the SPARC family of MCPs, that contains a domain arrangement different from its other family members and a SMOC-specific domain with no similarity to known moieties [14]. SMOC2 is a secreted protein that exhibits broad tissue distribution in the embryonic and adult mouse [14, 15], and is reported to be involved in various developmental processes [16–20]. SMOC2 is also strongly expressed during processes related to tissue remodeling, such as angiogenesis and response to chronic injury [21, 22]. The *SMOC2* gene is located on human chromosome 6 and is highly conserved across species. Variation in *SMOC2* copy-number is associated with human abnormalities, including hydrocephalus, brachycephaly, long face (vertical) and hypertelorism [23]. Patients with dentin dysplasia type I syndrome harbor point mutations in the *SMOC2* gene [24, 25]. The apparent role of SMOC2 in tissue development and remodeling correlates with its effects in cancer progression. In fact, SMOC2 has been shown to be important for metastasis in colon cancer [26], lung adenocarcinoma [27] and endometrial carcinoma [28], and proliferation in hepatocellular carcinoma [29]. To date, there are no reports that characterize SMOC2 in RCC.

Overall survival of metastatic cancer patients has not improved partly because predominant cancer treatments focus on the inhibition of cancer growth, with little emphasis on metastasis and less so on the influence of the ECM. Although there have been recent advances in diagnosis and treatment strategies, prognosis for metastatic RCC patients still remains poor since no proven therapeutic strategies have been discovered that efficiently forestall disease progression. Insights into the mechanisms of RCC metastasis and novel biomarkers for improved predictions are needed for clarification of RCC pathology and better clinical decisions. Herein we analyzed SMOC2 expression in RCC patient samples and cell lines, the influence of SMOC2 on EMT, and determined the effect that SMOC2 serves in RCC growth and metastasis in vitro and in vivo. Our study reveals a previously unrecognized role for SMOC2 in cancer progression by upregulating EMT features of RCC cells through integrin pathways and suggests that such regulation of cellular phenotype can lead to an increase in mitogenic and migratory behavior.

## Materials and methods

### Chemicals and reagents

Ponceau S, Ammonium Persulfate and TEMED were products of Millipore-Sigma Canada. Tween20, Tris-base, Glycine, EDTA, Methanol, SDS, Acrylamide and Bis-acrylamide were obtained from VWR, Canada. All chemicals were of ACS grade or higher.

### Ethics statement

The study conforms to the tenets of the Declaration of Helsinki, and approval of the human clinical protocol was obtained from the Maisonneuve-Rosemont Hospital Ethics Committee. All subject recruitment procedures and informed consent forms, including consent to use renal biopsy samples for research purposes, were approved by the Maisonneuve-Rosemont Hospital Ethics Committee and written informed consent was obtained from each patient. Renal biopsy specimens with sufficient tissue for immunohistochemical evaluation after the completion of diagnostic workup were included.

### Subjects

Deidentified human kidney tissue samples from patients with RCC (*n* = 4) were obtained from the Department of Pathology at HMR. Paraffin-embedded tissues were cut into 4-6-μm sections and processed for immunofluorescence and H&E staining.

### Immunofluorescence

Following antigen retrieval in citrate solution at pH6, sections were labeled with anti-SMOC2 (1:100; R&D Systems, USA) and anti-Vimentin 1:1000 (Millipore-Sigma). Slides were subsequently exposed to donkey anti-mouse Cy3-conjugated secondary antibody (1:200; Jackson ImmunoResearch Laboratories, USA) and donkey antirabbit AF647-conjugated (1:200 Jackson ImmunoResearch Laboratories). Fluoroshield with DAPI (Millipore-Sigma) was used for nuclear staining and mounting. All images were analyzed through NIH ImageJ using a color threshold algorithm (identical threshold settings for compared image sets) written by Gabriel Landini (version v1.8) available at http://www.mecourse.com/landinig/software/software.html.

### In vivo studies

#### Animal model

Male and female SCID (NOD SCID gamma) mice aged 8–12 weeks were used in this study with a sample size of *n* = 5–6, which were randomly divided into their respective groups. These mice were housed in a pathogen-free environment within the Maisonneuve-Rosemont Hospital Research Center animal facility and fed Harlan Teklad rodent diet (#2018 Envigo, QC, CAN) and water ad libitum. All experiments were conducted according to the Canadian Council on Animal Care guidelines for the care and use of laboratory animals, and under the supervision and approval of our local animal care committee, Comité de protection des animaux du Centre Intégré Universitaire de Santé et de Services Sociaux (CIUSSS) de l’Est-de-l'île-de-Montréal (Approved Protocol #2020-2042).

#### SMOC2 lentiviral transduction

For preparation of lentiviral particles, HEK293T cells were seeded at 50% confluency in a 15 cm dish one day before transfection. 12 µg of plasmid pUltra-Chili-Luc (Addgene #48688) with 2.5 µg of pMD2.G (Addgene #12259) and 7.5 µg psPAX2 (Addgene #12260) were co-transfected using Lipofectamine 2000 in OptiMEM (Gibco, Canada). Medium was changed 6 h after transfection. Virus supernatant was collected 48 h later and filtered with 0.45 µm PVDF syringe filter. 7 mL of lentivirus-containing medium supplemented with 15% FBS and polybrene at 4 µg/mL was used to transduce ACHN and 786-O cell lines. After 16 h of transduction, the media was replaced by fresh medium. After 72 h of transduction, dtomato positive cells were sorted by flow cytometry. A second round of transduction was performed with pLK0.1-puro (Addgene #8453 / Control) or pLenti-C-mGFP-P2A-Puro-SMOC2 (Origene #RC211979L4 / SMOC2). Positive clones were selected with puromycin treatment at 2 µg/mL for 72 h.

#### In vivo injections

786-O and ACHN cells transduced with a Luciferase plasmid as well as a control or SMOC2 plasmid were harvested, counted and washed twice with PBS. For the intravenous injections, male and female SCID mice were injected via tail vein with 500 000 cells in PBS. Tumor formation and metastasis were monitored by a fluorescent imager (IVIS Lumina III LT, PerkinElmer). Mice were sacrificed 20 days following injection. For orthotopic tumor cell implantations, SCID mice were placed in the prone position and an incision was made on the left flank. The left kidney was partially exteriorized. Then, a syringe (28 G) was used to inject 200 000 cells in PBS directly into the kidney. For this, the needle was inserted half of its length (5–6 mm) through the lower pole and the cell suspension was slowly injected. After removing the needle, gentle pressure was applied to the injection site for 60 s using a moistened cotton swab to avoid backflow of the tumor cell suspension. The muscle layer and the skin were closed. At the end of the procedure, mice received a subcutaneous injection of Butorphanol for pain relief. Mice, previously anesthetized with isoflurane, were euthanized by cervical dislocation 3- (786 cells) or 4- (ACHN cells) weeks after cell implantation. Tumor formation and metastasis were monitored by a fluorescent imager (IVIS Lumina III LT, PerkinElmer).

#### In vivo imaging

The substrate D-luciferin was injected into the intraperitoneal cavity of mice at a dose of 150 mg/kg body weight (30 mg/mL luciferin), approximately 5 min before imaging. Mice were anesthetized with isoflurane and placed on the imaging stage. Side, ventral and/or dorsal images were collected for 1 min of exposition using the IVIS Lumina III LT (Perkin Elmer) and Living Image software version 4.7.3 (Perkin Elmer). At day of sacrifice, mice were anesthetized and euthanized by cervical dislocation, organs were removed, washed in PBS and imaged. Primary tumor kidney tissue, lower extremity bones and lungs were harvested for histological evaluation.

### In vitro studies

#### Cell culture and media

786-O, ACHN HK-2 and MDCK cells were originally obtained from ATCC (USA). 786-O cells were maintained with (RPMI Gibco, Canada), supplemented with 1 mM sodium pyruvate (Gibco), 10.01 mM HEPES (Calbiochem, Canada) and 25 mM D-glucose (EMD, Millipore-Sigma, Canada). ACHN cells were maintained with DMEM. (Gibco) MDCK were maintained with EMEM (Gibco) supplemented with 1% sodium pyruvate and 1% NEAA. HK-2 cells were maintained in DMEM/F12 (Gibco). All cell lines were supplemented with 10% FBS (Gibco) and maintained in a humidified 5% CO_2_ incubator at 37 °C. All the cell lines studied were not reported in the ICLAC register of commonly misidentified cell lines.

#### SMOC2 transfection and recombinant protein treatment

##### Overexpression

Cells were transfected with 2 µg of Myc-SMOC2 (Origene, #Cat RC211979) or Myc-empty vector using JetPrime transfection reagent (Polyplus, VWR Canada) in a 6-well dish following manufacturers protocol for 24-72 h. Myc-empty vector was prepared in-house using the Myc-SMOC2 vector by restriction enzyme digestion to remove the SMOC2 fragment.

##### Silencing

Cells were seeded in a 6-well dish with 80 pmol of either ssiRNA or SMOC2 siRNA (SantaCruz, USA) using JetPrime transfection reagent (Polyplus; VWR, Canada), following manufacturer protocol for 6 h, followed by a 5 ng/mL treatment with TGFβ (Preprotech NJ, USA) for 15-20 h.

##### Recombinant protein

Cells were plated overnight in 6-well dish and treated with 10 ng/mL of recombinant SMOC2 protein (R&D Systems #Cat 5140-SM-050) or PBS vehicle diluted in fresh media. Media was replaced every 24 h with fresh recombinant protein or vehicle for the duration of the experiment.

##### Immunoprecipitation analysis

Cells were transfected with 2 µg of Myc-SMOC2 or Myc-empty vector were homogenized in IPH buffer (Thermo Fisher Scientific, 50 mM Tris [pH 8], 150 mM NaCl, 0.5% NP40) containing 1× protease and phosphatase inhibitor cocktail (Roche Applied Science) and left on a rocking platform for 1 hour. Protein concentration was determined using pierce BCA protein assay (Thermo Fisher Scientific). Between 800 μg to 1 mg of protein was incubated with anti-MYC beads (Sigma-Aldrich) overnight at 4 ^o^C. After centrifugation, the supernatant was removed and the beads were washed 4 times with 500 μL of IPH wash buffer (50 mM TRIS buffer, 5 mM EDTA, 150 mM NaCl, 0.5% NP-40). The beads were then boiled in 2X sample buffer for 6 min, followed by centrifugation, and used for Western blot analysis.

#### Western Blotting

Cells were harvested with ice-cold RIPA buffer (Thermo Fisher Scientific) containing protease and phosphatase inhibitors (Roche Life Science, Canada). Protein concentration was measured with BCA assay (Pierce, Thermo Fisher Scientific Samples of 10 to 50 μg of protein were separated by electrophoresis on a 10% gel and were electrophoretically transferred onto nitrocellulose membranes (Amersham Protran 0,45 μm, GE Healthcare Life science, Canada). Membranes were saturated with 5% NFDM in Tris buffered saline (TBS) containing 0.1% Tween20 (TBST) and washed with TBST. The following primary antibodies were used to detect specific proteins: SMOC2 (1:500, Abcam, #Cat ab56088), Myc (1:1000, Cell Signaling Technology #Cat 2276), E-cadherin (1:1000, Abcam # Cat ab15148), Fibronectin (1:5000, Abcam #ab23750), α-Smooth Muscle Actin (1:2000, Sigma #Cat A2547), Vimentin (1:3000, Santacruz, #Cat Sc-6260), GAPDH (1:5000, Abcam #ab8485), and Integrin Antibody Sampler Kit (1:1000, Cell Signaling Technology #Cat 4749). Horseradish peroxidase–conjugated secondary antibodies against mouse (Santa Cruz sc-516102) and rabbit (Santa Cruz sc-2357) were used to detect the appropriate primary antibodies. Bands were detected with the Clarity Max Western ECL Substrate from Bio-Rad Laboratories (Hercules, USA). Results were analyzed by computer-assisted densitometry using ImageQuant LAS-4000 system from GE Healthcare Life Sciences (Mississauga, CAN), ImageJ and FUJIFILM MultiGauge V3.0.

### Functional assays

#### Proliferation assay

Cells previously transfected or treated were lysed, seeded into 24 or 96 wells plates and were serum deprived (1% serum in respective media). Proliferation was measured as described in ref 11 with minor modifications. After 3 h of serum starvation, culture media was carefully aspirated and replaced with 100uL of fresh complete media, then 20 μl of a 5 mg/mL MTT solution was added to each well and the plate was incubated at 37 °C for 3,5 h. Thereafter the medium was aspirated and replaced with 150uL of MTT solvent (4 mM HCl, 0.1% NP40 in isopropanol). The plate was placed on a shaker for 30 min at 37 °C. After the formazan crystals had dissolved, the absorbance was measured at 540 nm with a reference wavelength of 630 nm on an ELx808 microplate reader (BioTek Instruments Inc., USA).

#### Migration assay

Serum starved treated cells were seeded into 8 μm cell culture inserts (Falcon, #Cat 353097) in 24-well culture plates (Falcon). Media with 5% FBS was added to the lower chamber as a chemoattractant. Assay plates were transferred to a 37°C incubator and let undisturbed for 24 h. Cells that had invaded the surface of the filter were fixed with Formalin 10%, (Hemochem) and stained with Crystal Violet 0.5% in 25% Methanol, (BDH). Inserts with stained cells were imaged at HMR imaging facility and quantified using ImageJ.

#### Scratch assay

Cells were grown to a semi confluent monolayer and were mechanically scratched (wound) using a standard 200 μL pipette tip. Suspension cells were washed away with media. Along the scratch, prefixed points were selected for representative photographs at 0 h and 24 h after initialization of the wound using a phase-contrast microscope.

Scratch assay was also performed using the Incucyte Live Cell SX5 system (Sartorius Canada, ON) as specified in manufacturer’s instructions. Briefly, 786-O cells were transduced with SMOC2 (vSMOC2) or empty vector, cultured and plated at different densities in triplicate, in an ImageLock 96 well plate. Cells were serum starved for 2 h before scratches were made in the monolayer of cells. A wound maker (Sartorius Canada, ON) was used to create identical scratches in the evenly distributed population. Plate was photographed every hour for 43 h. Data was analyzed with the Incucyte software. This system measures scratch closure in real time and automatically calculates wound confluence and density.

#### Morphology analysis

Cell size and branching were calculated using ImageJ. For cell size, cell bodies were outlined and calculated for their volume. For cell branching, a line was drawn at the point where the average cell morphology was cobblestone-like and another line was drawn at the point where the average cell extension had migrated. The distance between both lines determined the amount of cellular branching from the confluent cell layer.

### Statistical analysis

Results are expressed as mean ± standard error. Statistical significance for multiple comparisons was calculated by a Student’s t-test, and a *P*-value < 0.05 was considered statistically significant. Statistical analyses were performed with GraphPad Prism v6.07.

## Results

### SMOC2 is strongly upregulated in human RCC clinical samples and cell lines

**Fig. 1:**
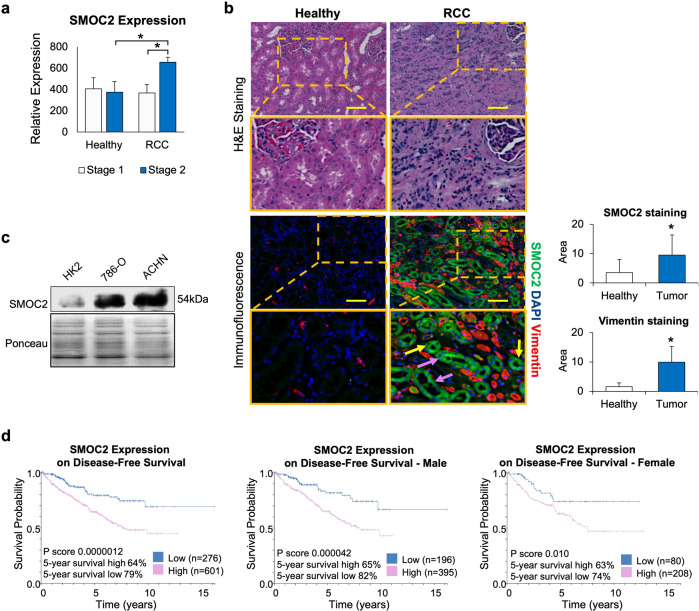
SMOC2 is upregulated in RCC epithelial cells and its high expression predicts poor RCC patient outcome. **a** SMOC2 mRNA expression in RCC patient biopsies at stage 1 and 2 compared to the healthy patients (*n* = 10 patients/condition). **b** H&E staining of RCC patient tissue biopsies. RCC tissue show loss of kidney structures, replaced by condensed cells. Immunofluorescence of RCC patient tissue biopsies show SMOC2 (green), vimentin (red) and DAPI (blue). Distal tubules are indicated by yellow arrows, while proximal tubules are indicated by purple arrows. Images are representative of *n* = 4; *p < 0.05 determined by t-test. Scale bar: 50 μm; 20X Magnification. **c** Endogenous SMOC2 levels in a normal human kidney cell line (HK2), and human RCC epithelial cell lines 786-O and ACHN. Ponceau staining served as a loading control. **d** Kaplan-Meier plot of SMOC2 mRNA expression levels (low vs. high) correlating patient survival rate. 5-year survival rates for low and high SMOC2 expression, p score and patient size are listed for total male and female patients.

To assess if SMOC2 expression is upregulated in RCC, we analyzed a microarray profile of tissue samples derived from 10 patient-matched normal and RCC tissue (Stage 1, *n* = 5; and Stage 2, *n* = 5; Accession number GSE-6344) [30]. From this analysis, we identified SMOC2 mRNA as significantly upregulated in Stage 2 RCC (Fig. 1a). To confirm with proteomic expression, immunostaining for SMOC2 in patient- healthy margins and Stage 2-3 RCC tissue revealed a significant increase in SMOC2 protein in biopsies that were positive for the RCC mesenchymal marker vimentin and H&E staining of solid tumor growth (Fig. 1b). Specifically, we detected SMOC2 in cells exclusively localized within both the epithelial proximal and distal tubules. Consistent with these patient data, SMOC2 is more highly expressed in cell lines derived from human RCC epithelial cells, ACHN and 789-O, than in cell lines derived from immortalized normal human kidney cells, HK-2 (Fig. 1c).

### High SMOC2 expression correlates with poor RCC patient survival

In order to investigate the prognostic value of SMOC2 expression in RCC patients, we examined the association of SMOC2 expression with disease-free survival of 877 RCC patients using sequencing results from the published database Protein Atlas (Fig. 1d) [31]. A Kaplan–Meier survival analysis revealed that patients with elevated expression of SMOC2 in renal tumors negatively correlated with disease-free survival. The 5-year survival of patients with high- versus low-SMOC2 expression was 64% and 79%, respectively. Similar results were seen between male and female patients. Overall, patients in the SMOC2 high group had significantly poorer survival than those in the SMOC2 low group (Fig. 1d). Therefore, high SMOC2 expression has an unfavorable clinical prognosis for RCC patients.

### SMOC2 promotes the morphology and expression profile of EMT

**Fig. 2:**
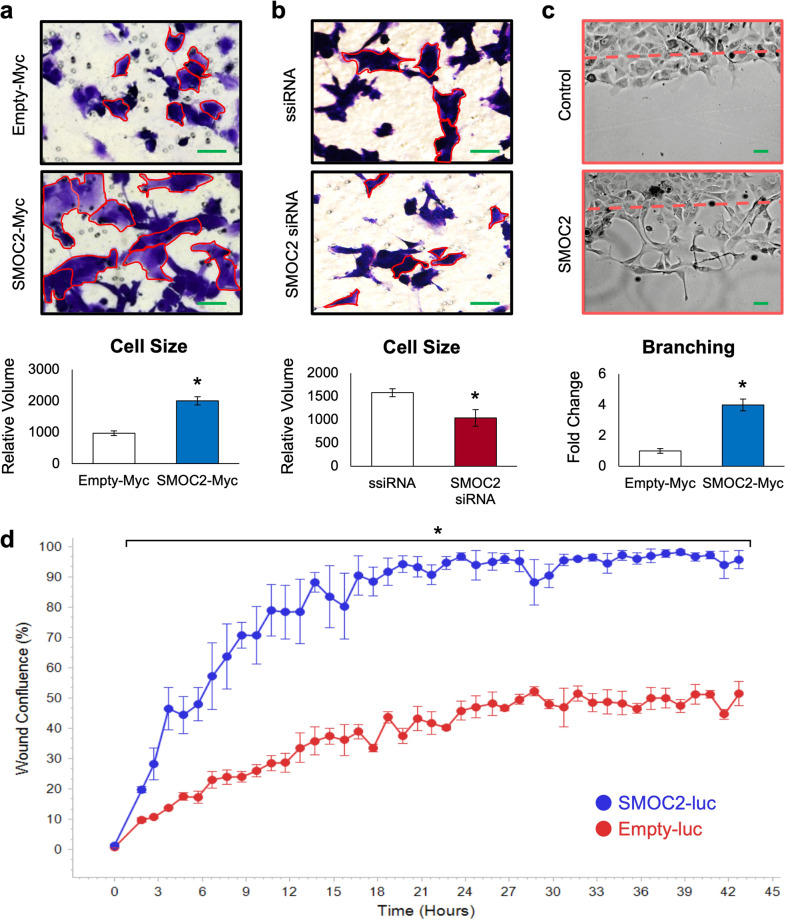
SMOC2 affects the morphology and colony organization of epithelial cells. ACHN cells were transfected for 24 h with **a** an empty-Myc or SMOC2-Myc vector, and **b** silencing RNA (ssiRNA) or SMOC2 siRNA, then evaluated for their morphology. siRNA transfections were cotreated with TGFβ1. Cells were outlined in red for increased visibility. **c** After 24 h of transfection with an empty-Myc or SMOC2-Myc vector, MDCK cells formed a confluent monolayer then scratched for a Scratch assay. Images of MDCK cells were taken 24 h after the inflicted scratch. **d** 786-O cells were transduced with a SMOC2-luc or empty-luc vector, which were used to form a confluent monolayer, then scratched for a Scratch assay. Live cell analysis was performed on such 786-O cells over a 43 h period to calculate the percentage of wound confluence as a function of time for triplicates (15 000 cells/well). Wound confluence (%) represents the fractional area of the wound that is occupied by cells. **a**, **b** were taken at 20X magnification and **c** was taken at 10X magnification. Scale bar: 50 μm. Images are representative of *n* = 3; *P < 0.05 determined by t-test between SMOC2 and control cells at each time point.

To begin to understand the implications of SMOC2 upregulation in tubular epithelial cells during RCC development, we examined by phase-contrast microscopy the in vitro effect of SMOC2 on epithelial cell lines. We found that SMOC2-Myc transfection of ACHN RCC cells was able to induce a voluminous cell body with elongated pseudopodia extensions, typical of a mesenchymal morphology, when compared to the narrow cell body and shorter extensions of empty-Myc control cells (Fig. 2a). The opposite occurred when SMOC2 was silenced in ACHN cells. Using TGFβ1 (5 ng/mL) as an EMT stimuli [32], SMOC2-siRNA transfection of ACHN cells were unable to achieve a mesenchymal morphology as seen by scrambled siRNA (ssiRNA) transfected cells (Fig. 2b). A scratch assay of SMOC2-Myc transfected MDCK epithelial cells revealed additional EMT hallmarks, such as cells losing both its normal features of cell-to-cell contact and apical-basal polarity to branch off into individual cells (Fig. 2c). This is in stark contrast to the epithelial phenotype found in our control, which included MDCK cells with a cobblestone morphology as they remain assembled into a defined and organized structure at the leading edge of the scratch (Fig. 2c). A scratch assay was also performed on 786-O RCC cells transduced with a vSMOC-luc or empty-luc vector. Over every hour of a 43 hr period, SMOC2 stimulated a very significant amount of wound migration than its control counterpart as observed by the percentage of wound confluency (Fig. 2d). We extended our analysis of wound confluency using various cell concentrations as well as analyzing their relative wound densities to confirm the significant influence SMOC2 has in closure of a scratch assay (Suppl. Fig. 1).

**Fig. 3:**
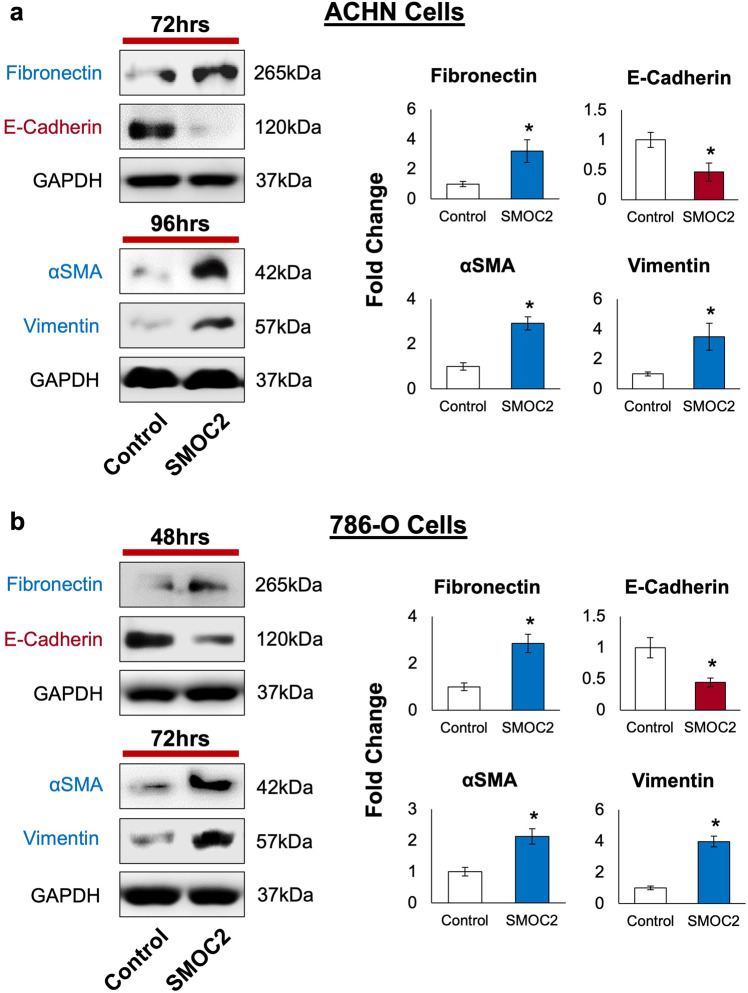
Recombinant SMOC2 treatment of RCC cells induces the protein expression of key EMT markers. **a** ACHN and **b** 786-O cells were treated with either 10 ng/mL recombinant SMOC2 or vehicle (control), then protein harvested at indicated times. Cell extracts were subjected to Western blot analysis for fibronectin, E-cadherin, αSMA and vimentin. GAPDH immunoblotting served as a loading control. Representative of *n* = 3; *P < 0.05 determined by t-test.

**Fig. 4:**
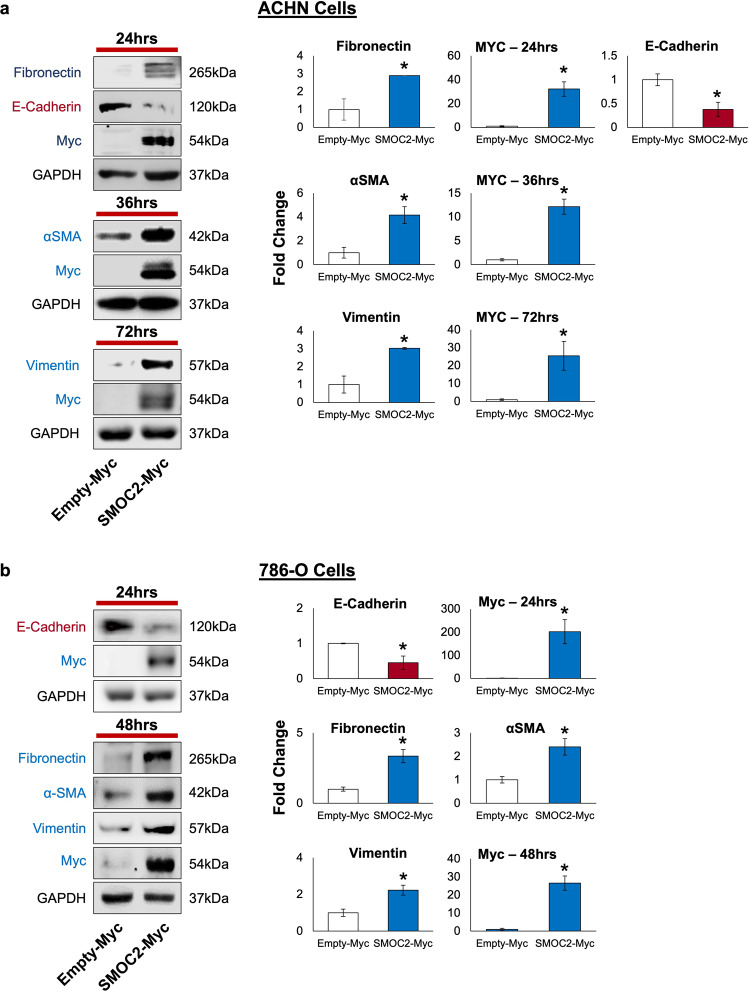
SMOC2 overexpression induces EMT markers in RCC cells. **a** ACHN and **b** 786-O cells were transfected with either a SMOC2-Myc or empty-Myc vector, then protein harvested at indicated times. Cell extracts were subjected to Western blot analysis for fibronectin, E-cadherin, αSMA, vimentin and SMOC2 (Myc). GAPDH immunoblotting served as a loading control. Representative of *n* = 3; *P < 0.05 determined by t-test.

On the basis that SMOC2-treated epithelial cells experience a mesenchymal phenotype, we decided to analyze if SMOC2 can induce the protein expression of EMT markers in RCC cell lines ACHN and 786-O. Given that SMOC2 is a secreted protein, we treated ACHN and 786-O with recombinant SMOC2 (10 ng/mL) and analyzed their protein expression for markers of EMT at various time points. Consistent with its phenotypic induction of EMT, SMOC2 treatment of both RCC cell lines initiated with a loss in the epithelial cell marker E-cadherin, followed by an intense increase in mesenchymal markers fibronectin, alpha-smooth muscle actin (αSMA) and vimentin when compared to their respective controls (Fig. 3a, b). Furthermore, transfection with an SMOC2-Myc vector revealed this EMT protein profile to be concomitant with the appearance of SMOC2 overexpression (Fig. 4a, b). Therefore, SMOC2-treatment and -overexpression show the possibility of SMOC2 having a paracrine and autocrine effect when eliciting EMT in kidney tubular cells.

**Fig. 5:**
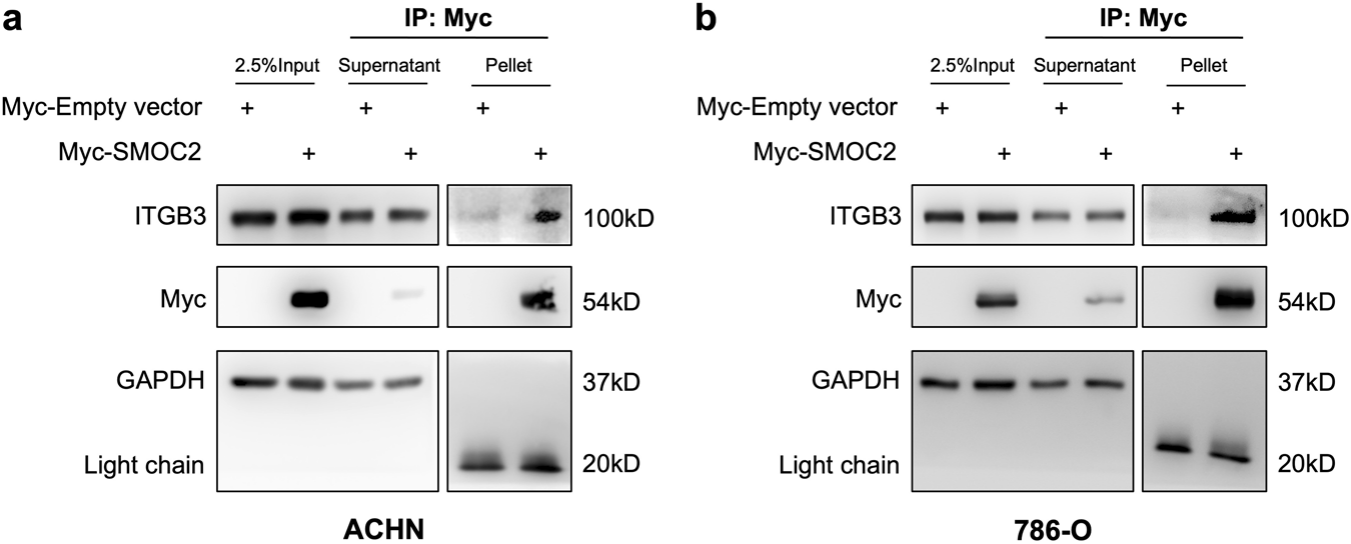
SMOC2 binds to integrin β3. **a** ACHN and **b** 786-O cells were transfected with either a SMOC2-Myc or empty-Myc vector, then protein harvested after 24 h. Cell extracts were immunoprecipitated for Myc. Western blot analysis was performed on whole cell extracts (2.5% Input), supernatant and Myc-immunoprecipitated samples for Myc, integrin β3 (ITGB3) and GAPDH. Western blot images are representative of repeated experiments.

**Fig. 6:**
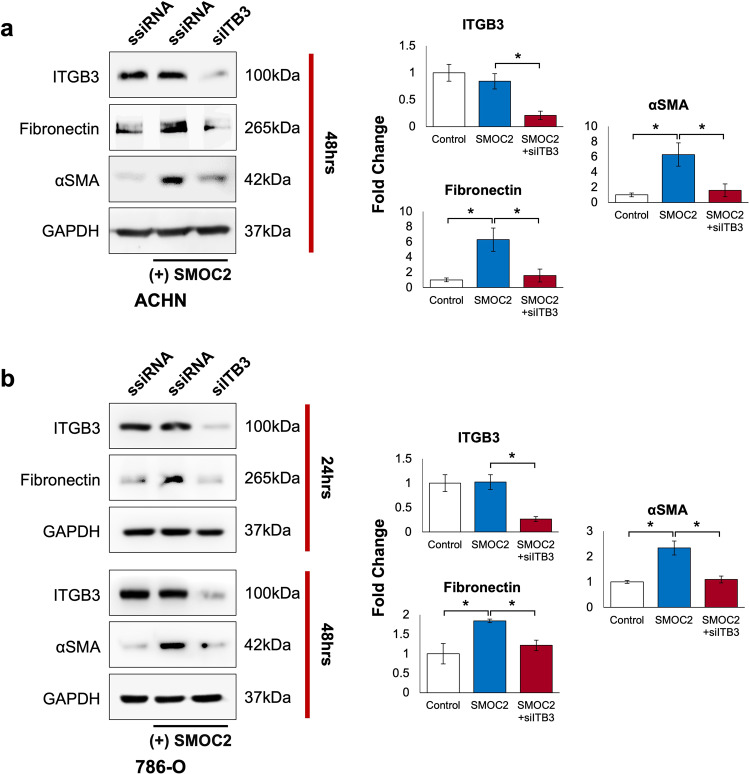
SMOC2 interacts with integrin β3 to mediate EMT. **a** ACHN and **b** 786-O cells were transfected with either scrambled siRNA (ssiRNA) or integrin β3 siRNA (siITB3) 24 h prior to 5 ng/mL SMOC2 recombinant protein treatment, then protein harvested after 24 and 48 h. Western blot was performed on whole cell extracts for integrin β3 (ITGB3), and EMT markers fibronectin and αSMA. GAPDH immunoblotting served as a loading control. Images are representative of *n* = 3; *P < 0.05 determined by t-test.

**Fig. 7:**
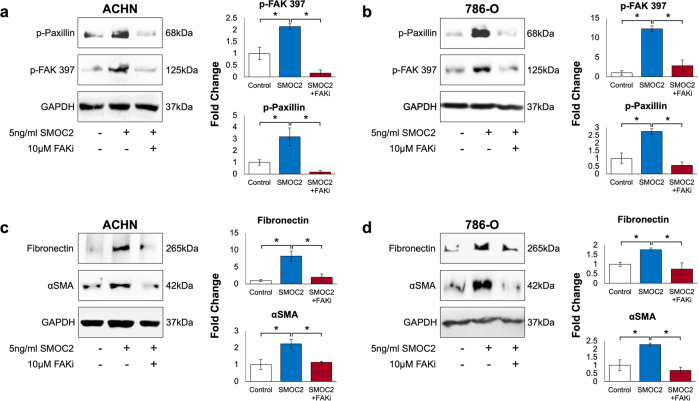
SMOC2 activates EMT through integrin proximal proteins. **a**, **c** ACHN and **b**, **d** 786-O cells were pretreated for 2 h with a focal adhesion kinase inhibitor (10 μM FAKi), then treated with recombinant SMOC2 or vehicle (control). After 5 min of SMOC2 treatment, protein from **a** ACHN and **b** 786-O cells was harvested and a Western blot analysis was performed on whole cell extracts for phosphorylated Focal Adhesion Kinase (P-FAK) at site Y397, and phosphorylated paxillin at site Tyr118. After 24 h of SMOC2 treatment, protein from **c** ACHN and **d** 786-O cells was harvested and a Western blot analysis was performed on whole-cell extracts for ECM proteins Fibronectin and αSMA. GAPDH immunoblotting served as loading controls. Images are representative of *n* = 3; *P < 0.05 determined by t-test.

The integrin pathway has been extensively shown to promote the EMT phenotype in several settings [33–37]. We and others have shown SMOC2 to interact with the cell through integrin β1 binding in fibroblasts [22], and αv, β1 and β6 binding in keratinocytes [15], as well as activating the integrin proximal molecule Focal Adhesion Kinase (FAK) [18, 22, 26]. To verify their interaction with integrin, we transfected ACHN and 786-O cells with a SMOC2-Myc or empty-Myc vector and whole-cell extracts were Myc immunoprecipitated, then the pull-down was blotted for various integrin subtypes. The results provide a two-cell type analysis that SMOC2 binds integrin β3 from RCC epithelial cells (Fig. 5a; Suppl. Fig. 2a). We extended our results to show SMOC2 mediates its effects on EMT through integrin β3 by transfecting ACHN and 786-O cells with integrin β3-siRNA (siITG3) or ssiRNA for 24 h, then treating them for an additional 24-48 h with recombinant SMOC2. After confirming that integrin β3 expression could be silenced in both cell models, we showed that siITG3 prevented SMOC2 from inducing the expression of our EMT markers fibronectin and αSMA when compared to their respective controls (Fig. 6a, b). Moreover, we show that the same recombinant SMOC2 treatment of ACHN and 786-O cells that activated EMT also triggered the activation of the integrin pathway by phosphorylating its downstream targets FAK and paxillin (Fig. 7a, b; Suppl. Fig. 2b), which could be blocked with a pretreatment of a FAK inhibitor. Pretreatment of the FAK inhibitor also prevented SMOC2 from activating fibronectin and αSMA expression (Fig. 7c, d).

Together, our results suggest that SMOC2 signals through the integrin pathway of RCC cells to express the molecular markers EMT.

### SMOC2 affects the proliferation and motility of RCC cells

**Fig. 8:**
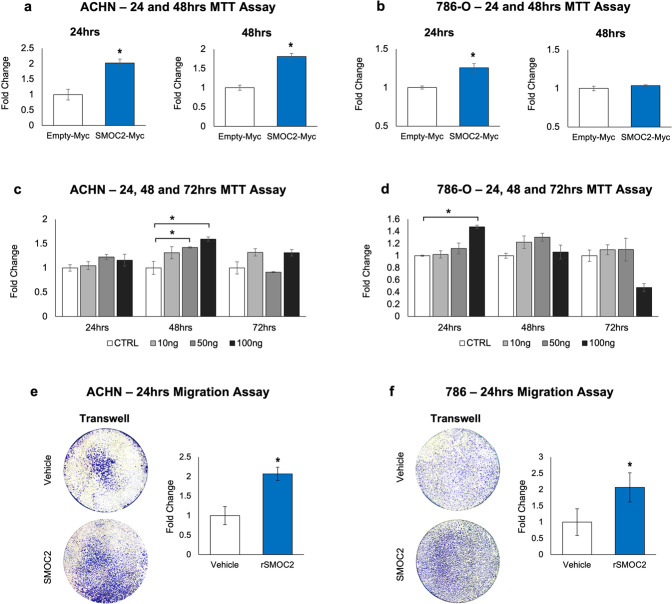
SMOC2 activates the common properties of EMT. Viability of **a** ACHN and **b** 786-O cells transfected with either a SMOC2-Myc or empty-Myc control vector, and **c** ACHN and **d** 786-O cells treated with either vehicle (CTRL) or 10, 50, 100 ng/mL SMOC2 were measured over time by an MTT assay. A Boyden chamber assay was performed on **e** ACHN and **f** 786-O cells treated 24 h with vehicle or 10 ng/mL SMOC2. Images are representative transwells. Each experiment was performed with an *n* = 3; *P < 0.05 determined by t-test.

The loss in cell-to-cell contact combined with the loss in epithelial polarity and the generation of pseudopodal extensions are coordinated EMT processes that permit cancer cells to disassemble, and migrate and survive within tissue sites away from their primary tumor sites [38]. Given that these phenotypic features of EMT were induced by SMOC2, we studied the likelihood that RCC cells exposed to SMOC2 may acquire the ability to migrate and possibly be more viable, both of which are features of successful metastasis. Using an MTT assay in the absence of serum, SMOC2-Myc overexpression in ACHN and 786-O cells resulted in higher viability rates over the course of 48 h than their empty vector controls (Fig. 8a, b). Similar viability results were obtained through recombinant SMOC2 treatment of ACHN and 786-O cells, although both cell lines had different temporal patterns between them (Fig. 8c, d). We also found that recombinant SMOC2 treatment of such RCC cell lines was more effective than vehicle-treated controls in promoting migration in a Boyden chamber assay (Fig. 8e, f).

Collectively, our results suggest that SMOC2 affects RCC cells by promoting phenotypic changes associated with EMT in order to prime them for EMT-related functions, as shown by SMOC2 induction of survival and migration.

### Downregulation of SMOC2 interferes with the EMT process of RCC cells

**Fig. 9:**
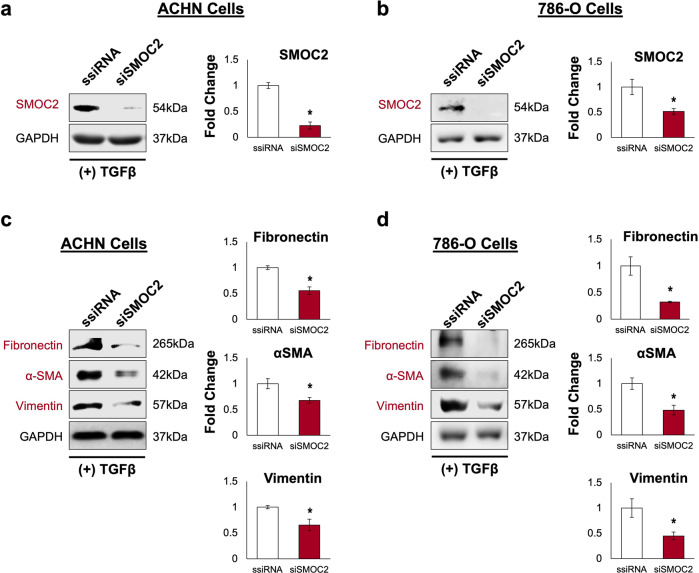
SMOC2 is an important component in transforming epithelial cells into mesenchymal cells. After 24 h of treatment with SMOC2 siRNA (siSMOC2) or scrambled siRNA (ssiRNA), **a**, **c** ACHN and **b**, **d** 786-O cells were treated with 5 ng/mL TGFβ1 for an additional 24 h. Western blot analysis was performed on whole cell extracts for **a**, **b** SMOC2, and **c**, **d** fibronectin, αSMA and vimentin. GAPDH immunoblotting served as a loading control. Representative of *n* = 3; *P < 0.05 determined by t-test.

**Fig. 10:**
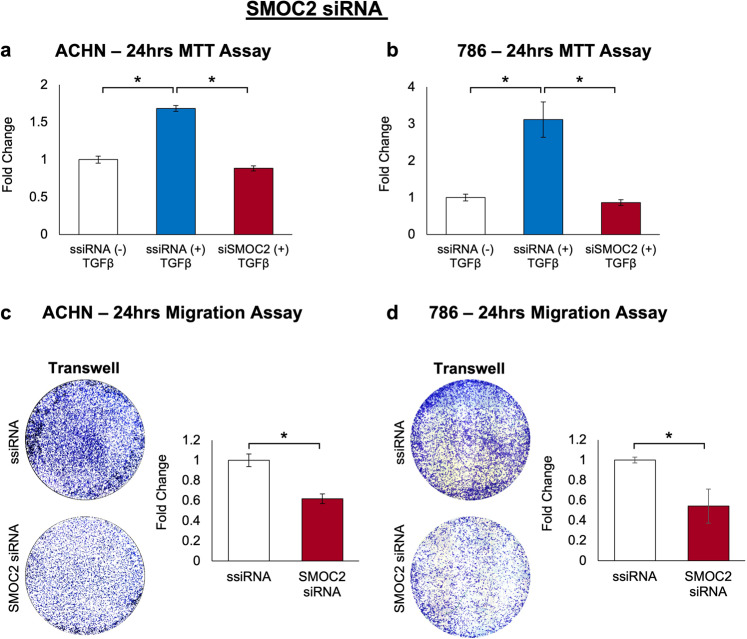
Silencing SMOC2 reduces EMT activities in RCC cells. After 24 h of treatment with SMOC2 siRNA or scrambled siRNA (ssiRNA), **a** ACHN and **b** 786-O cells were treated with 5 ng/mL TGFβ1 for 24 h. Viability was evaluated using an MTT assay. Boyden chamber assay was performed on **c** ACHN and **d** 786-O cells treated 24 h with SMOC2 siRNA or ssiRNA. Images are representative transwells. Each experiment was performed with an *n* = 3; *P < 0.05 determined by t-test.

To corroborate the above data and determine the level of importance SMOC2 has on the events of EMT in RCC cells, we transfected ACHN and 786-O cells with either a control scrambled siRNA (ssiRNA) or SMOC2 siRNA to reduce the expression of SMOC2 (Fig. 9a, b), and monitored any potential changes in the induction of EMT by TGFβ1. Through Western blot analysis we observed that both RCC cells transfected with SMOC2 siRNA had lower levels in the EMT proteins fibronectin, αSMA and vimentin when compared to the control (Fig. 9c, d). We also tested the effect of inhibiting the expression of SMOC2 on the EMT functions of migration and survival. Using TGFβ1 as a mitogenic stimuli, an MTT assay showed that ACHN and 786-O cells transfected with SMOC2 siRNA had reduced their viability when compared to their respective controls (Fig. 10a, b). Furthermore, the reduction of SMOC2 in both RCC cell lines had lower levels of migration within a Boyden chamber (Fig. 10c, d). Evidently, our SMOC2 downregulation studies indicate that the presence of SMOC2 is important for the occurrence of EMT migration and survival.

### SMOC2 promotes RCC tumor growth and metastasis in vivo

**Fig. 11:**
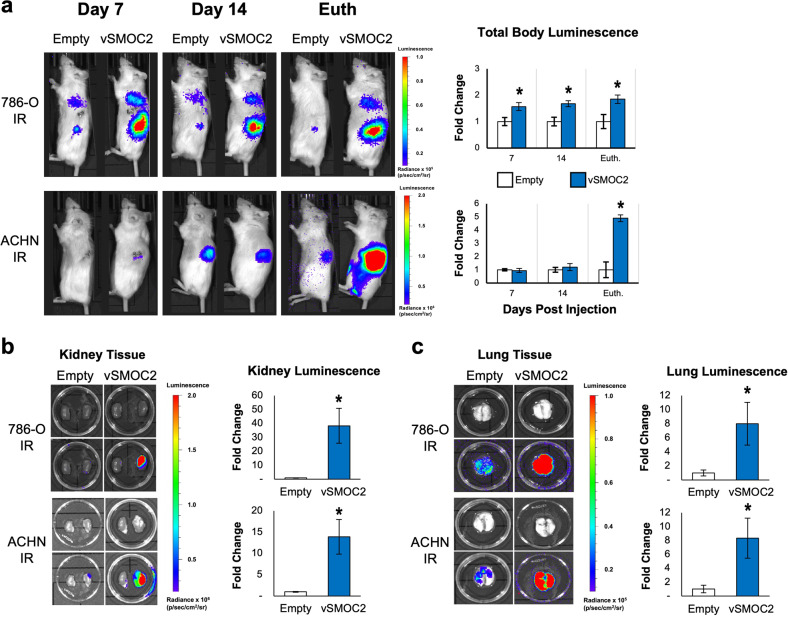
SMOC2 overexpression promotes RCC tumor growth and metastasis in vivo. **a** ACHN and 786-O cells were transduced with a luciferase-labeled SMOC2 (vSMOC2) or empty vector which were used for intrarenal (IR) injections of SCID mice. Mice were euthanized (Euth.) at 16 days for 786-O cell injections and 27-29 days for ACHN cell injections. Each experiment was performed with an *n* = 5–6; *P < 0.05 determined by t-test. Whole-body luminescence was detected by IVIS imaging, as shown by representative images. **b** Kidney and **c** lung tissue were isolated and tumor growth was quantified by luminescence as shown by representative images (Top row: brightfield image; Bottom row: superimposed top row brightfield image with luminescent image). Each experiment was performed with an *n* = 5-6; *P < 0.05 determined by t-test.

Given the in vitro impact that SMOC2 has on RCC survival and the characteristics of EMT, we next determined whether SMOC2 impacts RCC tumor growth and metastasis in vivo. We first prepared luciferase-labeled SMOC2 overexpressing (vSMOC-luc) and control (empty-luc) vectors and stably transfected them into ACHN and 786-O cells, then validated them for SMOC2 overexpression (Suppl. Fig. 3) and equal luciferase activity between vectors (Suppl. Fig. 4). After performing orthotopic tumor xenografts with intrarenal (IR) implantation of vSMOC-luc or empty-luc RCC cells, immunodeficient SCID mice were intraperitoneally injected with luciferin at various time points (Post-implantation days: 7, 14 and euthanized day) and luminescence intensity was detected to quantify primary tumor growth as well as potential systemic metastasis using the live IVIS imaging system. In both ACHN and 786-O cell implantation models, we observed a significant increase in primary RCC tumor growth in our whole-body imaging and isolated kidney tissues from our vSMOC-luc models as compared to their empty-luc controls (Fig. 11a, b), however only vSMOC-luc 786-O cells showed a significant difference at each time point (Fig. 11a). Furthermore, luminescent signals from harvested lung tissue showed that mice implanted with vSMOC-luc 786-O or ACHN cells had much more lung metastatic foci than their respective controls (Fig. 11c). Collectively, the results from our in vivo IR xenograph mouse models provide evidence that SMOC2 promotes RCC tumor cell growth as well as metastasis to the lungs.

**Fig. 12:**
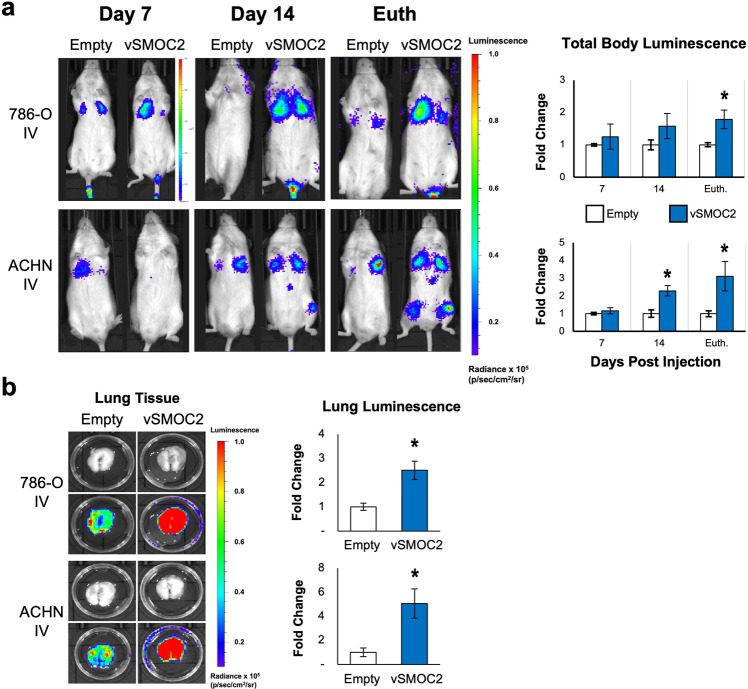
SMOC2 expression in RCC cells increases metastasis to the lungs in immunodeficient mice. **a** ACHN and 786-O cells were transduced with a luciferase-labeled SMOC2 (vSMOC2) or empty vector and injected intravenously into the tail vein of SCID mice, then euthanized (Euth.) at 17–18 days (both cell type injections). Each experiment was performed with an *n* = 5-6; *P < 0.05 determined by t-test. Whole-body luminescence was detected by IVIS imaging, as shown by representative images. **b** Lung tissue was isolated and tumor growth was quantified by luminescence as shown by representative images (Top row: brightfield image; Bottom row: superimposed top row brightfield image with luminescent image). Each experiment was performed with an *n* = 5-6; *P < 0.05 determined by t-test.

To substantiate our metastatic results, we performed intravenous tail injections of vSMOC-luc or empty-luc transfected ACHN or 786-O cells into immunodeficient SCID mice. On post-injection days 7, 14 and euthanized day, vSMOC-luc and empty-luc cells were monitored for dissemination by intraperitoneally injecting mice with luciferin and imaging them using a IVIS imaging system. Results show that both 786-O and ACHN SMOC2 overexpressing cells appeared at significantly higher levels in the lung area of whole-body images than controls (Fig. 12a), which were confirmed by luminescent signaling of isolated lung tissue (Fig. 12b). Interestingly, ACHN vSMOC-luc cells also appeared to localize in the hind limb bones at later time points (Fig. 12a). This was confirmed by luminescent signaling of isolated hind limb bones (Suppl. Fig. 5); however, a variation of signals between samples did not show a significant difference but a trend of SMOC2 overexpressing cells migrating to the area than controls.

## Discussion

As non-structural components of the ECM, MCPs have recently emerged as important components of the ECM to regulate matrix processing and a variety of cellular functions. Their expression is context dependent, particularly exhibiting high expression during the tissue remodeling processes of embryogenesis and development [12]. In adult tissues, MCPs are generally expressed at low levels but highly expressed in numerous diseases to participate in their pathologies [9, 10, 39, 40], including cancers [13]. This has prompted the targeting of MCPs in the development of therapeutic strategies, many of which have advanced into clinical trials [41].

A family of MCPs whose members are known for their tumorigenic roles is the SPARC family [13], particularly a member called SPARC. SPARC expression is significantly upregulated in cells and stroma from a number of cancers, including glioma, breast and cervical melanoma [42–45], with oncogenic roles in cell growth, invasion and survival. Although other members share this oncogenic potential, some inhibit cancer progression such as FSTL1 [13], which has been recently shown to interact with another MCP called osteopontin to inhibit lung cancer metastasis [46]. Over recent years, SPARC member SMOC2 was studied for its role in development and fibrosis, suggesting a possible function for this protein in cancer progression since these tissue-altering processes share key signaling pathways [47, 48]. As expected, SMOC2 was shown to be expressed in various solid cancers with an importance for several oncogenic roles [13]. Although we and others have identified SMOC2 expression in the developing and injured kidney [16, 22], there are no previous reports on the relationship between SMOC2 and RCC.

**Fig. 13:**
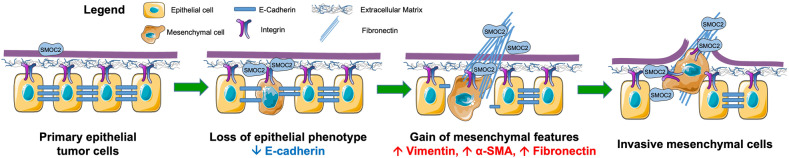
Schematic representation of SMOC2 induction of EMT markers and migration. SMOC2 binds to integrin of RCC cells and activates in a paracrine and autocrine fashion EMT by reducing E-cadherin intracellular binding for detachment, increasing αSMA cytoskeleton for larger cellular spreading, increasing pseudopodia extensions for migration, and increasing fibronectin expression for integration into migration tracks. Figure was produced using Servier Medical Art (http://smart.servier.com/).

In the present study, we highlight a novel role for SMOC2 in RCC. Using published gene expression profiles [30, 31], we identified SMOC2 as being significantly expressed in RCC tissue compared to normal renal tissue with a poor prognosis for RCC patients. More importantly, we show *de novo* protein synthesis of SMOC2 to be much higher in the tubular epithelial cells of patients with biopsy-proven RCC. Using RCC cell lines ACHN and 786-O, SMOC2 recombinant and enforced expression significantly increased EMT-markers with accompanying EMT-phenotypic and -metastatic features, which were abrogated by SMOC2 siRNA. As a secreted protein, SMOC2-treatment and -transfection also show their respective paracrine and autocrine potential on RCC cells, and that such effects are mediated through the integrin signaling pathway. Finally, we translated our findings in vivo through various xenograph models showing SMOC2 promoting tumor growth and metastasis. Taken together, this suggests that RCC tubular epithelial cells express and secrete SMOC2 into its microenvironment to stimulate their EMT and enhance their metastatic potential (Fig. 13).

The capability of cancer cells to undergo EMT requires changes in the expression of specific proteins involved in cell-to-cell interactions as well as in the cytoskeleton structure in order to perform several EMT functions, which evidently leads to a dramatic phenotypic transformation. In our case, we specifically showed that SMOC2 stimulated RCC cells to undergo EMT by decreasing E-cadherin expression, increasing fibronectin and αSMA expression, and developing pseudopodal extensions. The common accompanying function for each of these outcomes include the loss of E-Cadherin-mediated intercellular adhesion, an increase in fibronectin for potential migration tracks, an increase in αSMA-incorporation into the cytoskeleton for larger cellular spreading, as well as pseudopodal extensions for movement [49–52]. These corresponding functions are consistent with our Scratch assay data showing SMOC2 transfected cells branching off into individual cells from a cell layer sheet for cellular expansion and our Boyden chamber data where SMOC2 influenced RCC cells to migrate at a higher rate than their controls. We also showed that SMOC2 can increase cellular viability, a function that can promote the growth of EMT-RCC cells needed at their new metastasized location. Furthermore, we provide evidence that SMOC2 is an important component to promoting the phenotype and function of EMT in RCC since SMOC2 ablation significantly attenuated EMT protein markers and its capacity to proliferate and migrate. Collectively, such SMOC2-induced EMT events are frequently associated with the metastatic process of many epithelial tumors (e.g. kidney cancer) [53], which involves the detachment and extravasation of cancer cells from the primary site to their migration, invasion and proliferation at distant organs. In fact, we may associate such SMOC2-induced EMT effects with our observation that SMOC2 increases metastasis within our in vivo models. These findings are consistent with other cancer studies which have reported SMOC2 to be important for signaling EMT in colon cancer [26, 54], metastasis in lung adenocarcinoma [27] and colon cancer [26, 54], and proliferation in hepatocellular carcinoma [29], endometrial cancer [55] and colon cancer [26]. Furthermore, SMOC2 expression was found to be higher in the metastatic form of head and neck squamous cell carcinoma and canine mammary adenocarcinoma when compared to their primary form [56, 57].

The signaling pathways responsible for SMOC2 activity have been primarily focused on integrin-mediated pathways, which are known for their activation of EMT. Our current findings present SMOC2 binding to integrin β3 and phosphoactivating the integrin proximal proteins FAK and paxillin. In addition, this integrin pathway is responsible for SMOC2 stimulation of EMT in RCC cell lines, as shown through our integrin β3 siRNA and FAK inhibitor studies. Specifically, SMOC2 activated FAK by phosphorylating its tyrosine 397 (Y397) and 925 (Y925) residues and paxillin by phosphorylating its tyrosine 188 (Y118) residue (Fig. 7; Suppl. Fig. 2). Each of these phosphosites are consistent with our SMOC2 induction of RCC migration and pseudopodal extensions because Y397 phosphorylation was shown to be critical for the invasive properties of FAK in oral squamous cell carcinoma cells [58], Y118 phosphorylation on paxillin regulates cell migration in Nara Bladder Tumor II (NBT-II) cells [59], and Y925 phosphorylation of FAK is required for FAK-mediated cell migration and cell protrusion in mouse embryonic fibroblasts [60]. The involvement of the integrin pathway in SMOC2 signaling was also confirmed in the SMOC2 induction of metastasis using a colorectal cancer model [26].

Although research has advanced our understanding of RCC metastasis, no proven therapeutic strategies have been discovered that efficiently forestall disease progression. The role of SMOC2 in EMT may offer insight as a therapeutic target against metastasis; however, the mechanistic details and in vivo consequences of SMOC2-induced EMT for metastasis and viability of RCC cells needs to be further studied. From a biomarker perspective, we provide evidence that SMOC2 is a poor prognostic marker of RCC from RNAseq data. This is consistent with our SMOC2 staining in RCC tissue biopsies which were very contrasting to its near absence in normal tissue. Furthermore, as a secreted protein, increased expression of SMOC2 may shed off into the urine at earlier stages of RCC and reveal its translational potential as a non-invasive biomarker, as we have shown in fibrotic kidneys [22]. Therefore, our study on SMOC2 offers novel mechanistic insight about EMT metastasis in RCC and provides an important rationale to potentially monitor and target SMOC2 as a RCC biomarker and therapeutic.

## Supplementary information

SUPPLEMENTAL MATERIAL

Uncropped Western Blots

## Data Availability

All data generated or analyzed during this study are present in this published article. Uncropped Western blots are presented as Supplemental Information. Any additional information pertinent to the data may be requested from the corresponding author.
