# Supplementary material for: SMOC2 promotes an epithelial-mesenchymal transition and a pro-metastatic phenotype in epithelial cells of renal cell carcinoma origin
Source: Cell Death Dis. 2022 Jul 22;13(7):639. doi: 10.1038/s41419-022-05059-2 (PMC9307531; doi:10.1038/s41419-022-05059-2)
Supplement: Supplementary file 1 — SUPPLEMENTAL MATERIAL [file 41419_2022_5059_MOESM1_ESM.pdf]

Suppl. Fig. 1

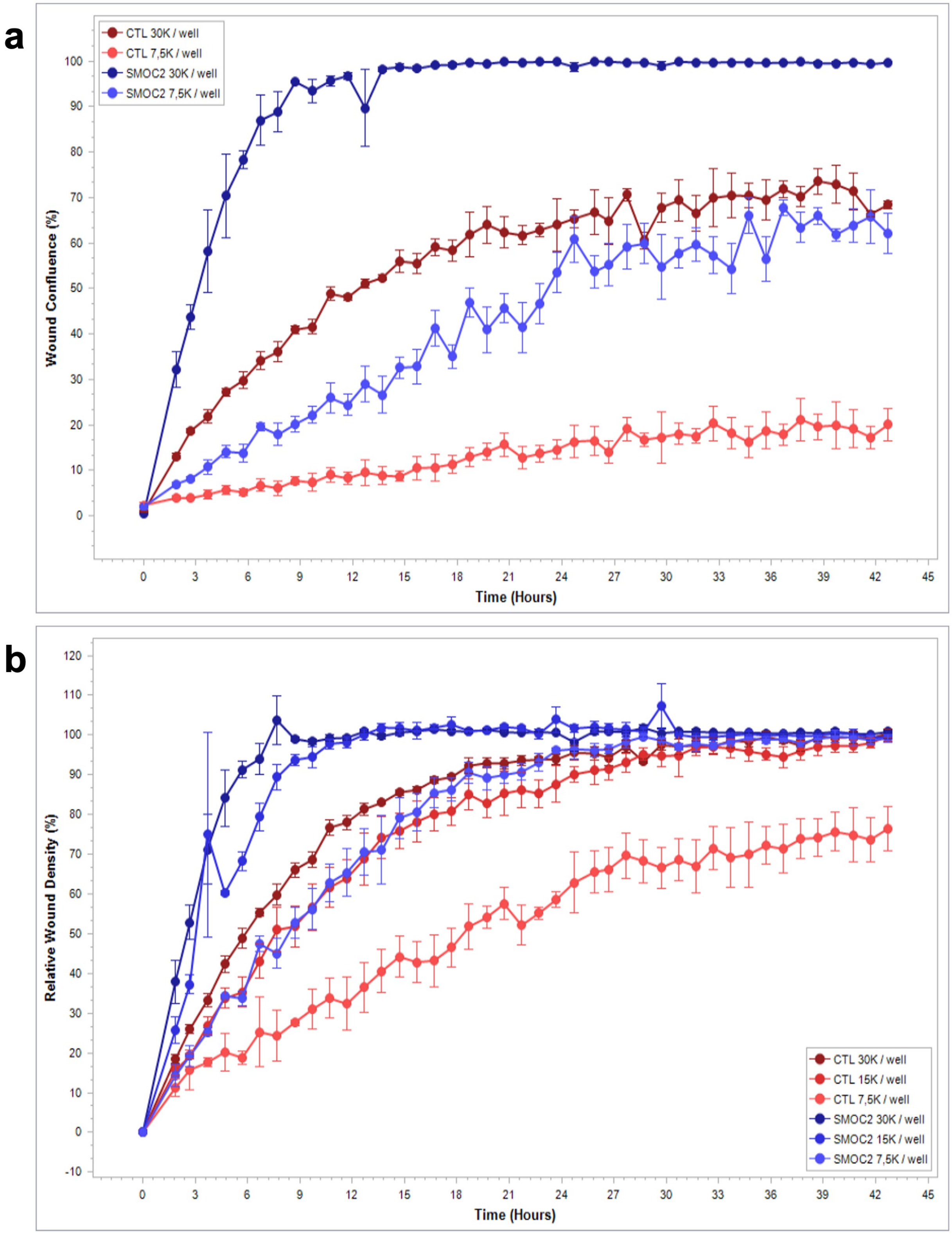
a
CTL 30K / well
CTL 7,5K / well
SMOC2 30K / well
SMOC2 7,5K / well
Wound Confluence (%)
Time (Hours)
b
Relative Wound Density (%)
CTL 30K / well
CTL 15K / well
CTL 7,5K / well
SMOC2 30K / well
SMOC2 15K / well
SMOC2 7,5K / well
Time (Hours)

Suppl. Fig. 1 SMOC2 overexpression promotes RCC wound closure. 786-O cells were transduced with an empty-luc or SMOC2-luc vector, which were used to form a monolayer then scratched for a Scratch assay. Live cell analysis of 786-O cells was monitored over a 43hr period for the percentage of **a** wound confluence and **b** relative wound density. Wound confluence (%) represents the fractional area of the wound that is occupied by cells. Relative wound density is a measure (%) of the density of the wound region relative to the density of the cell region. Each experiment was performed with an n=3; *$P < 0.05$ determined by t-test.

Suppl. Fig. 2

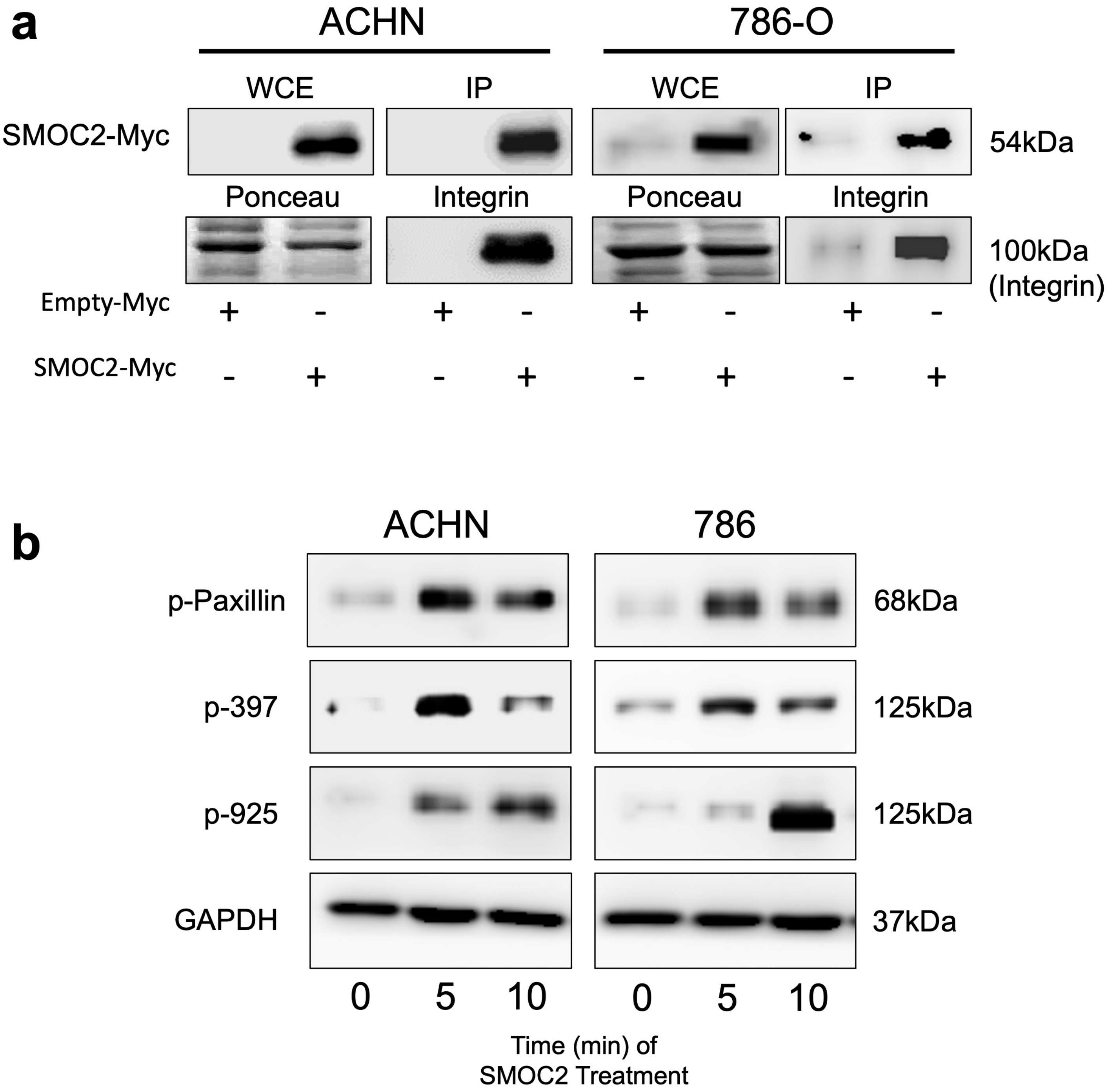
a
ACHN
786-O
WCE
IP
WCE
IP
SMOC2-Myc
54kDa
Ponceau
Integrin
Ponceau
Integrin
100kDa
(Integrin)
Empty-Myc
+ - + - + - + -
SMOC2-Myc
- + - + - + - +
b
ACHN
786
p-Paxillin
68kDa
p-397
125kDa
p-925
125kDa
GAPDH
37kDa
0 5 10 0 5 10
Time (min) of
SMOC2 Treatment

Suppl. Fig. 2 SMOC2 binds to integrin and activates its downstream pathway. a ACHN and 786-O cells were transfected with either a SMOC2-Myc or empty-Myc vector, then protein harvested after 24h. Cell extracts were immunoprecipitated for Myc. Western blot analysis was performed on whole cell extracts (WCE) and Myc-immunoprecipitated samples for Myc and integrin β3. Ponceau staining served as a loading control. **b** ACHN and 786-O cells were treated with either recombinant SMOC2 or vehicle (control), then protein harvested at indicated time points. A representative Western blot was performed on whole cell extracts for phosphorylated Focal Adhesion Kinase (P-FAK) at sites Y397 and Y927, and paxillin at site Tyr118. GAPDH immunoblotting served as a loading control.

Suppl. Fig. 3

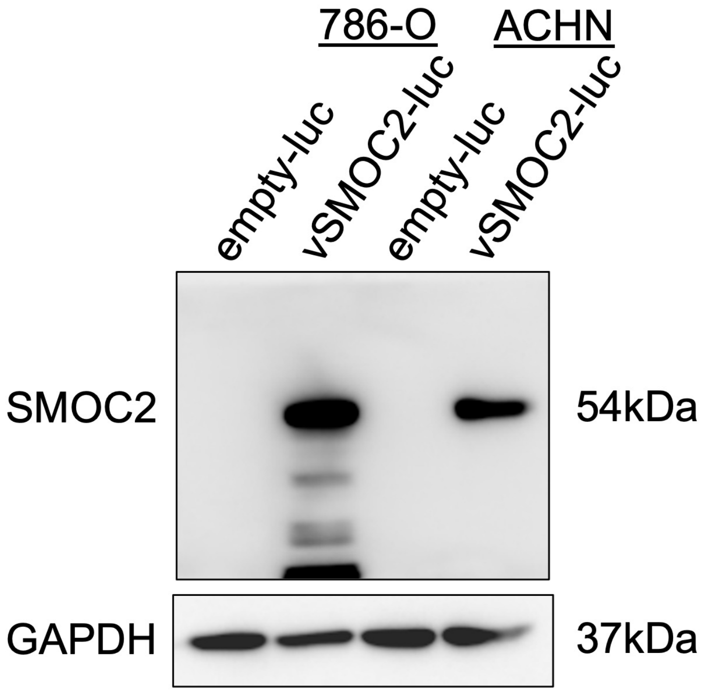

786-O
ACHN
empty-luc
vSMOC2-luc
empty-luc
vSMOC2-luc
SMOC2
54kDa
GAPDH
37kDa

Suppl. Fig. 3 SMOC2 expression in RCC cells. ACHN and 786-O cells were transduced with a luciferase-labeled SMOC2 (vSMOC2-luc) or empty vector. A representative Western blot was performed for the expression of SMOC2.

Suppl. Fig. 4

a

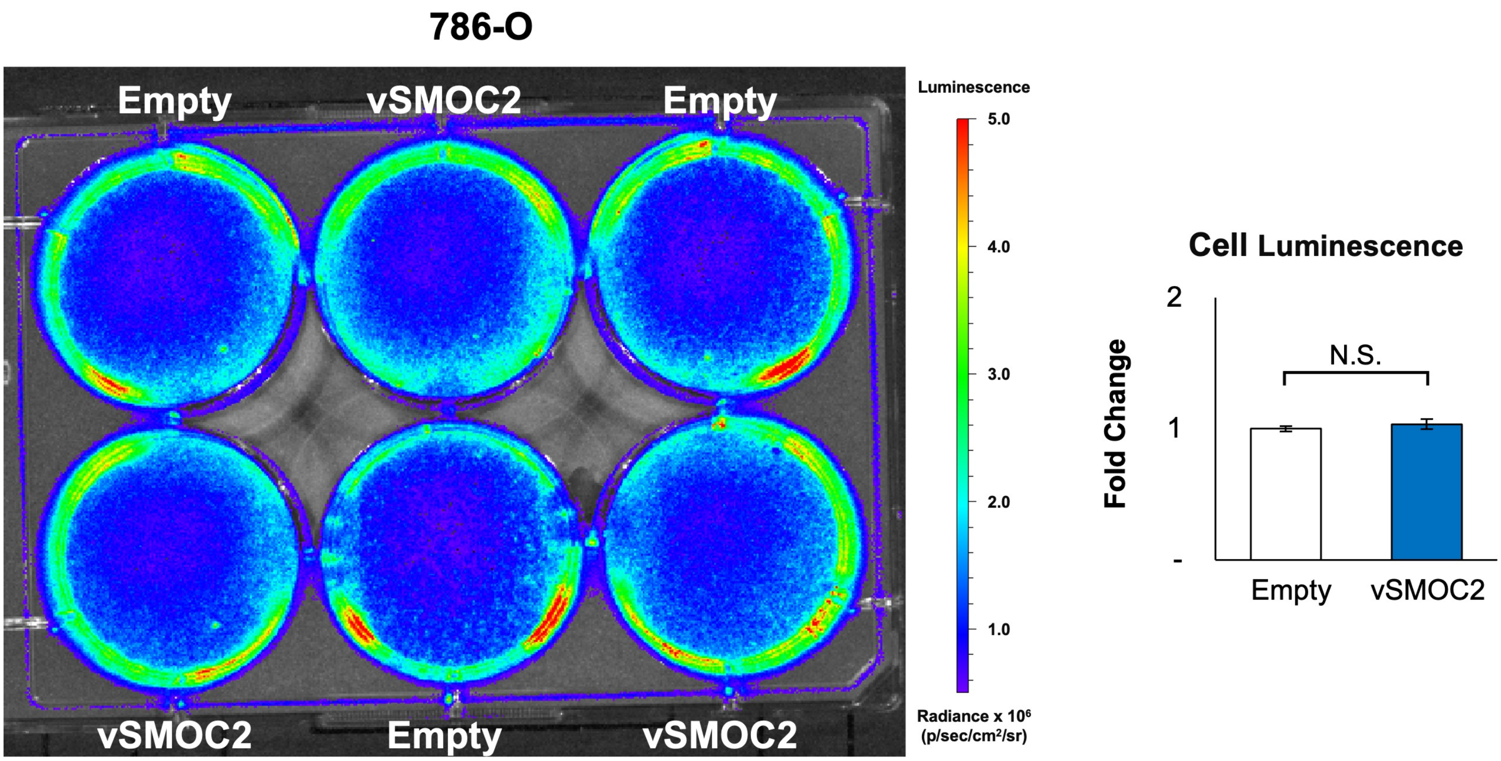

b

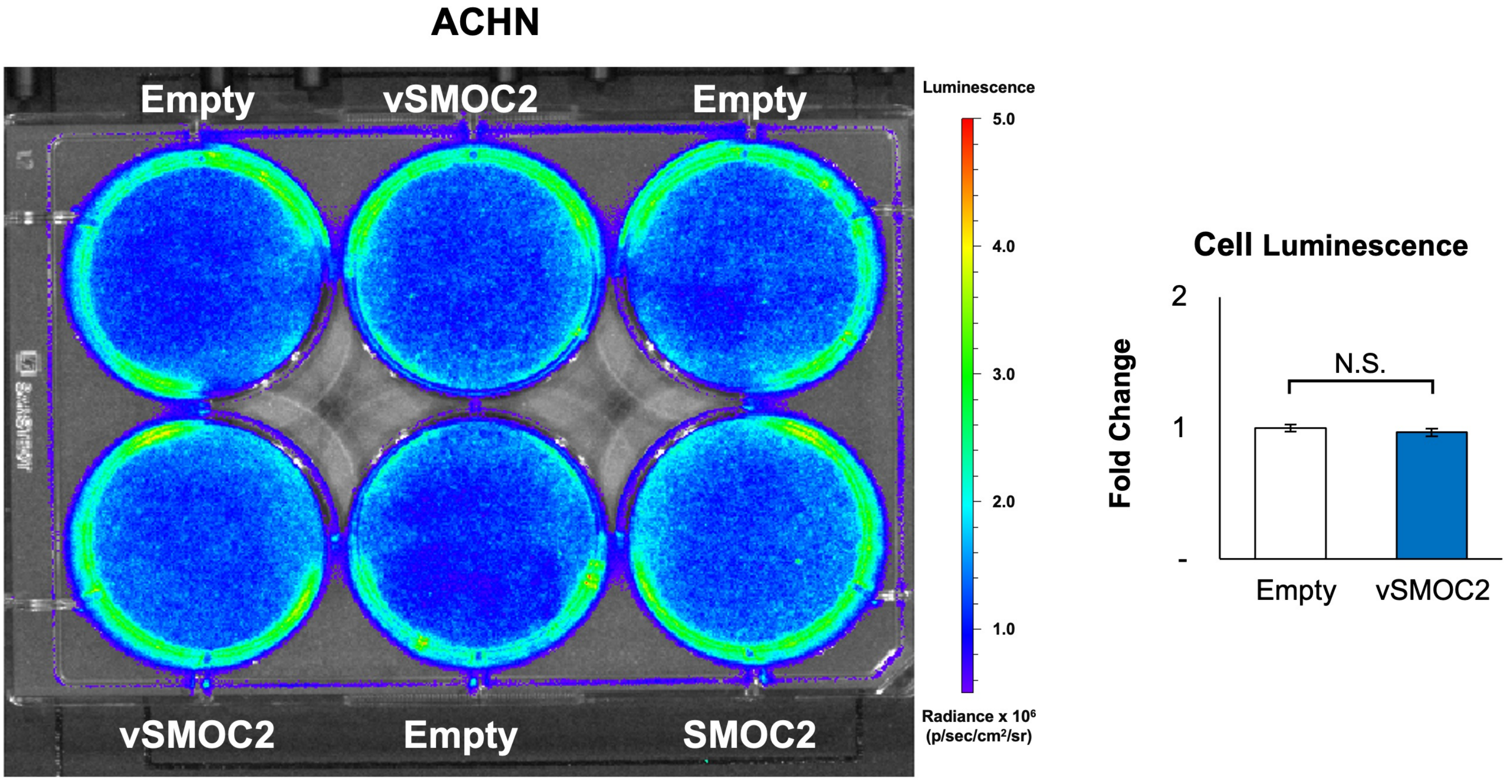

Suppl. Fig. 4 Luminescence in RCC cells. ACHN and 786-O cells were transduced with a luciferase-labeled SMOC2 (vSMOC2) or empty vector (Empty) which were used for our *in vivo* models. Cells were plated at **a** 400 000 cells/well (ACHN) or **b** 200 000 cells (786-O), and let to adhere for ~3h. Immediately before imaging, media was carefully removed and media containing 150 µg/mL D-Luciferin was added. Image was recorded 10 minutes following media change. Cell luminescence was detected by IVIS imaging; $*P < 0.05$ determined by t-test. N.S.: no significant difference.

Suppl. Fig. 5

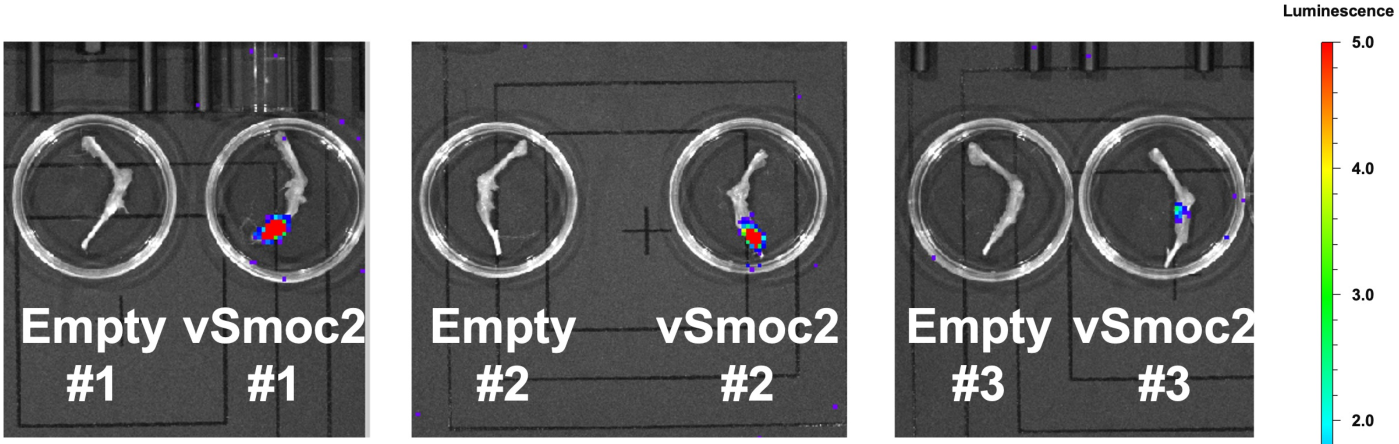

Luminescence
5.0
4.0
3.0
2.0
1.0
Radiance x 10⁴
(p/sec/cm²/sr)
Empty #1
vSmoc2 #1
Empty #2
vSmoc2 #2
Empty #3
vSmoc2 #3

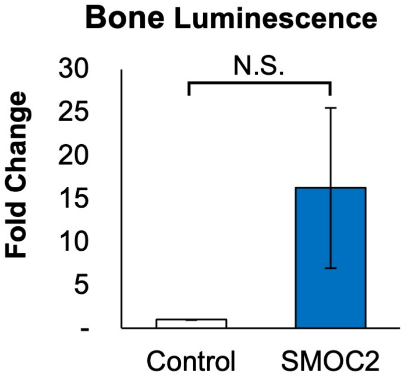

Bone Luminescence
Fold Change
30
25
20
15
10
5
-
N.S.
Control
SMOC2

Suppl. Fig. 5 SMOC2 expression in RCC cells increases metastasis to the hind limb bones in immunodeficient mice. ACHN cells were transduced with a luciferase-labeled SMOC2 (vSMOC2) or empty vector which were used to inject intravenously into SCID mice and euthanized at 17-18 days. Each experiment was performed with an n=5-6. The hind limb bones were harvested after intraperitoneally injecting mice with luciferin, and luminescence was detected by IVIS imaging, as shown by representative images. N.S.: no significant difference.
