# Supplementary material for: SMOC2 promotes an epithelial-mesenchymal transition and a pro-metastatic phenotype in epithelial cells of renal cell carcinoma origin
Source: Cell Death Dis. 2022 Jul 22;13(7):639. doi: 10.1038/s41419-022-05059-2 (PMC9307531; doi:10.1038/s41419-022-05059-2)

Fig. 1c

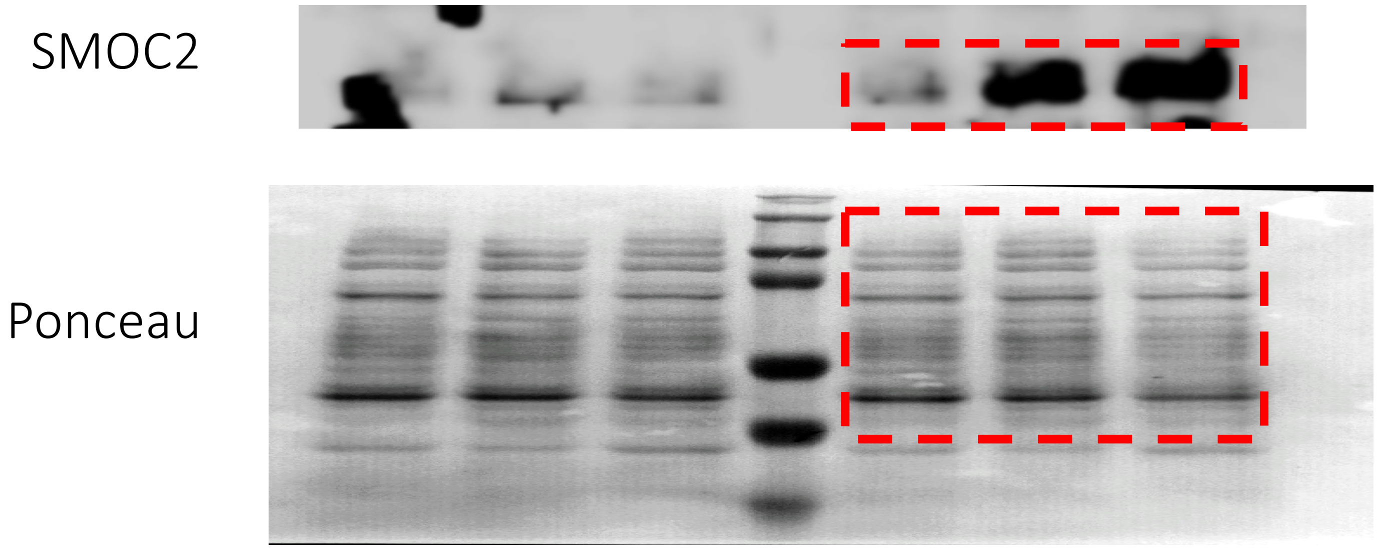

# Fig. 3a ACHN O/E – 24hrs

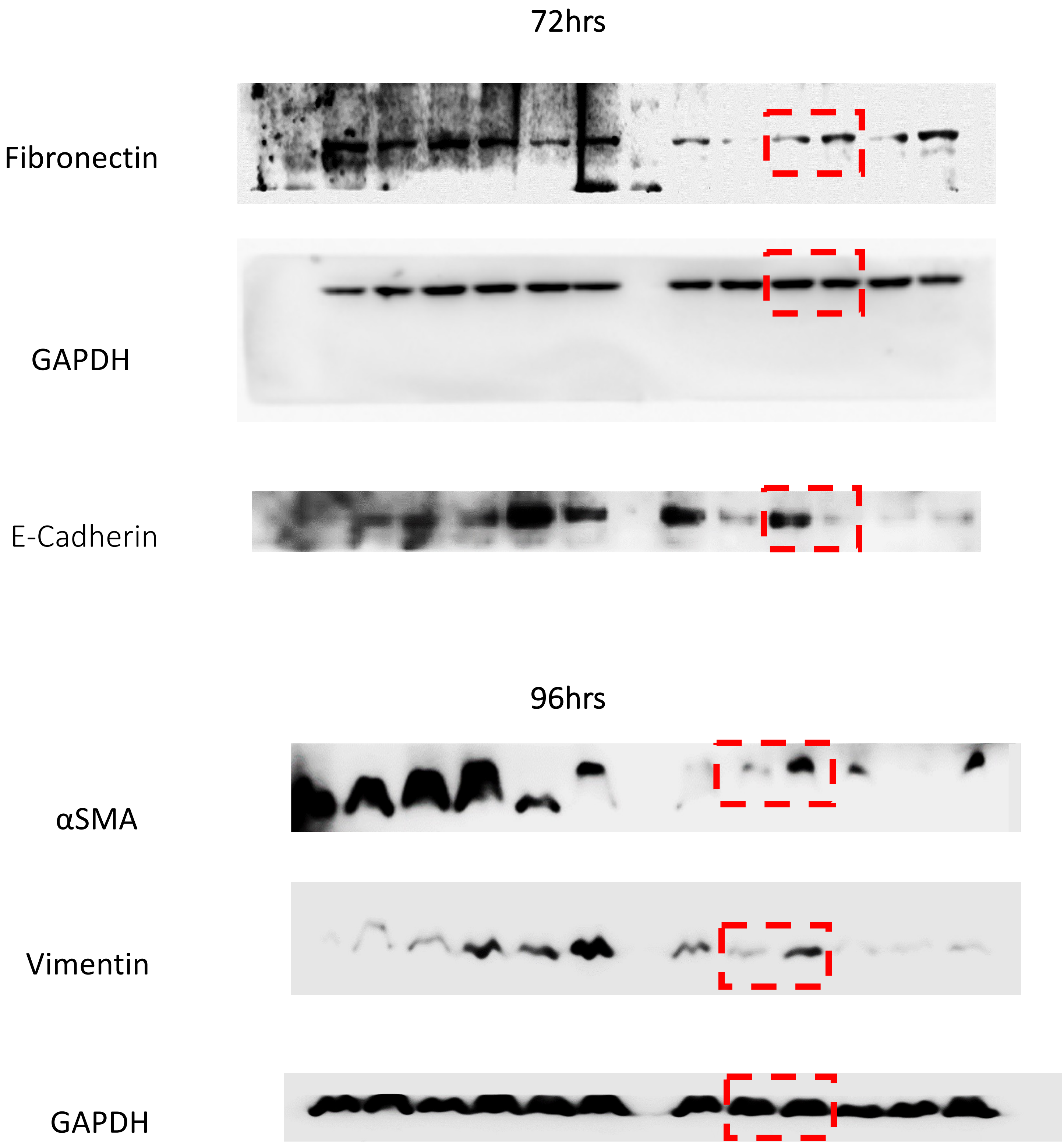

Fig. 3b 786-O O/E

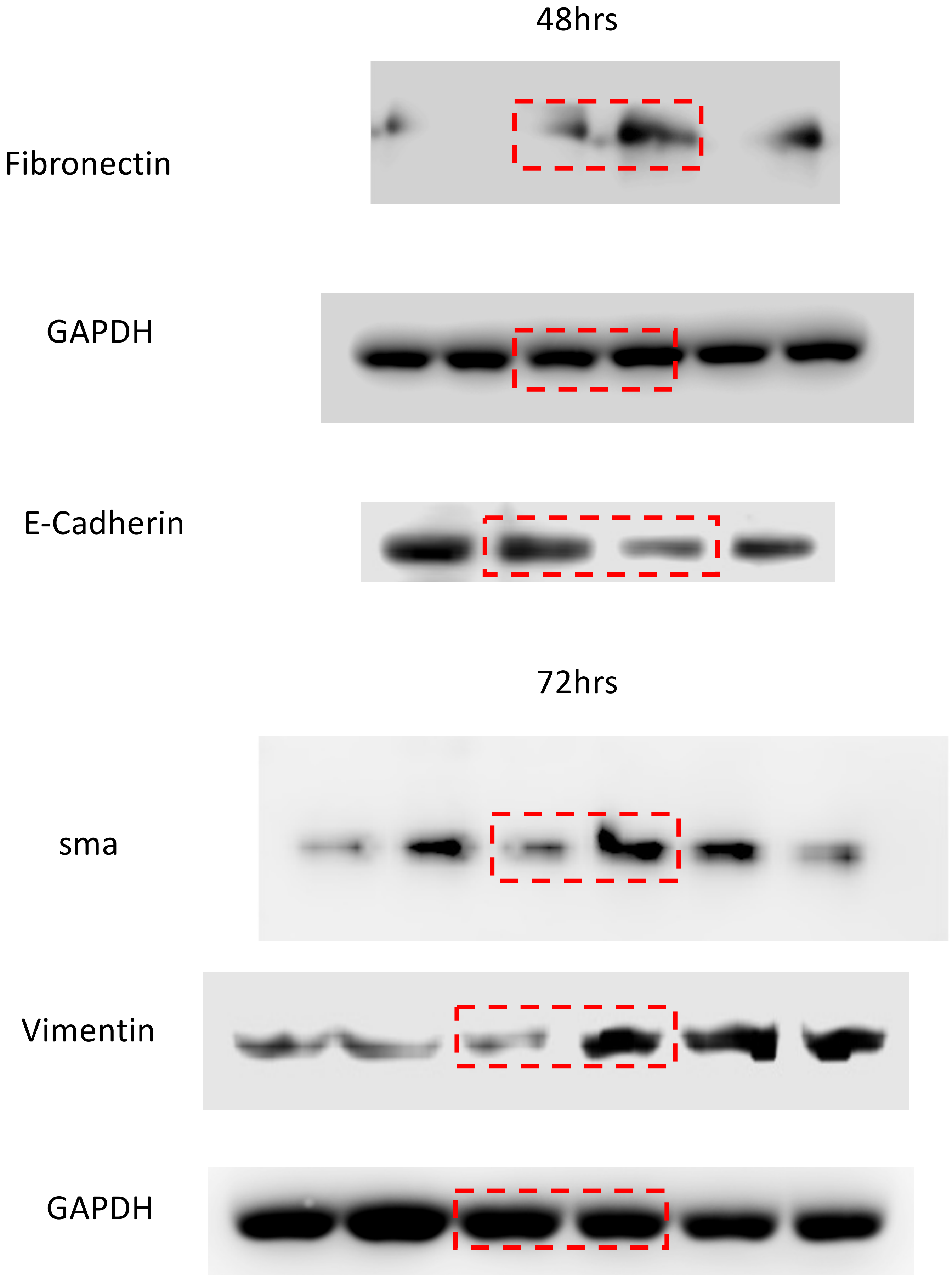

# Fig. 4a ACHN O/E – 24hrs

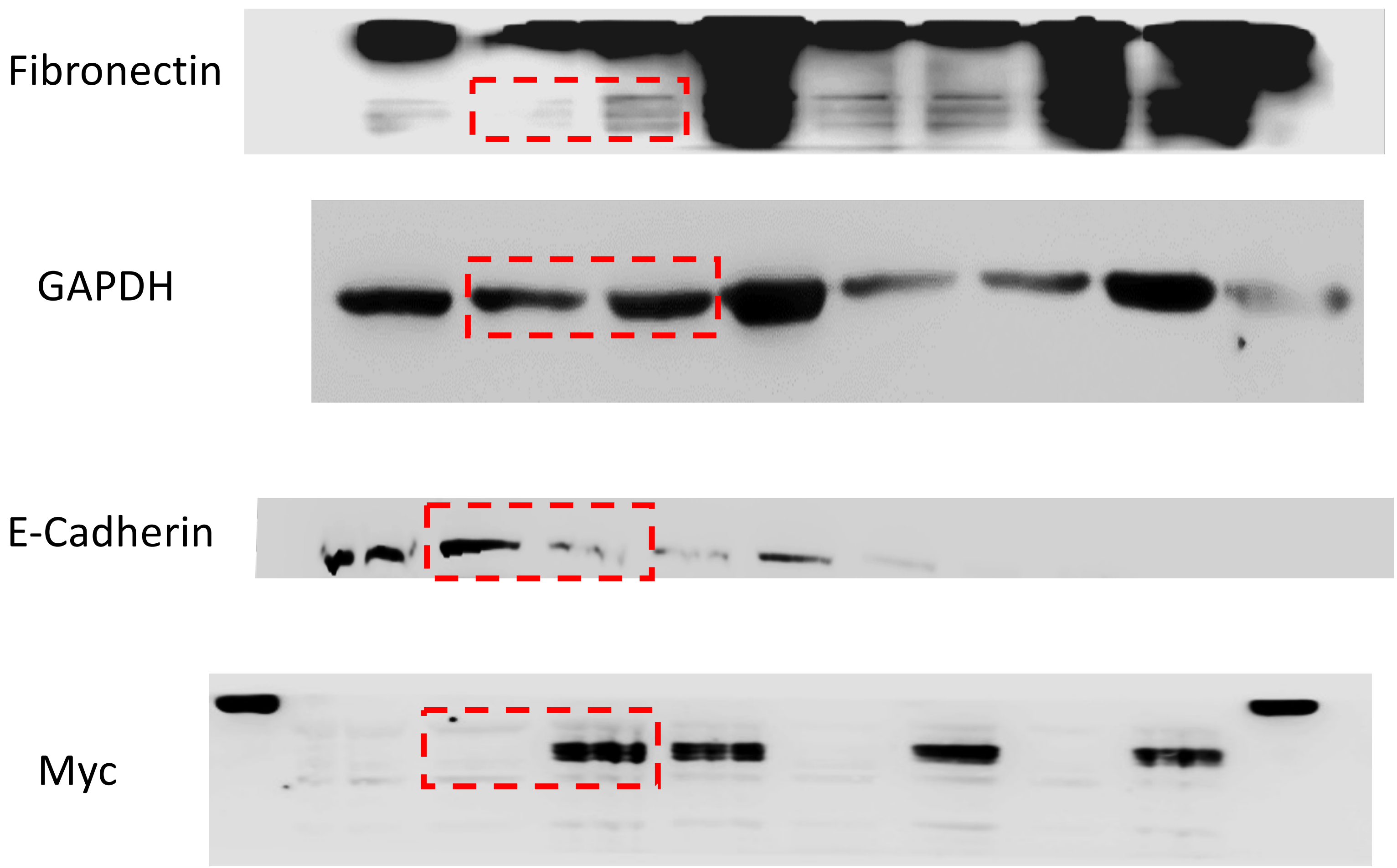

# Fig. 4a ACHN O/E – 36hrs

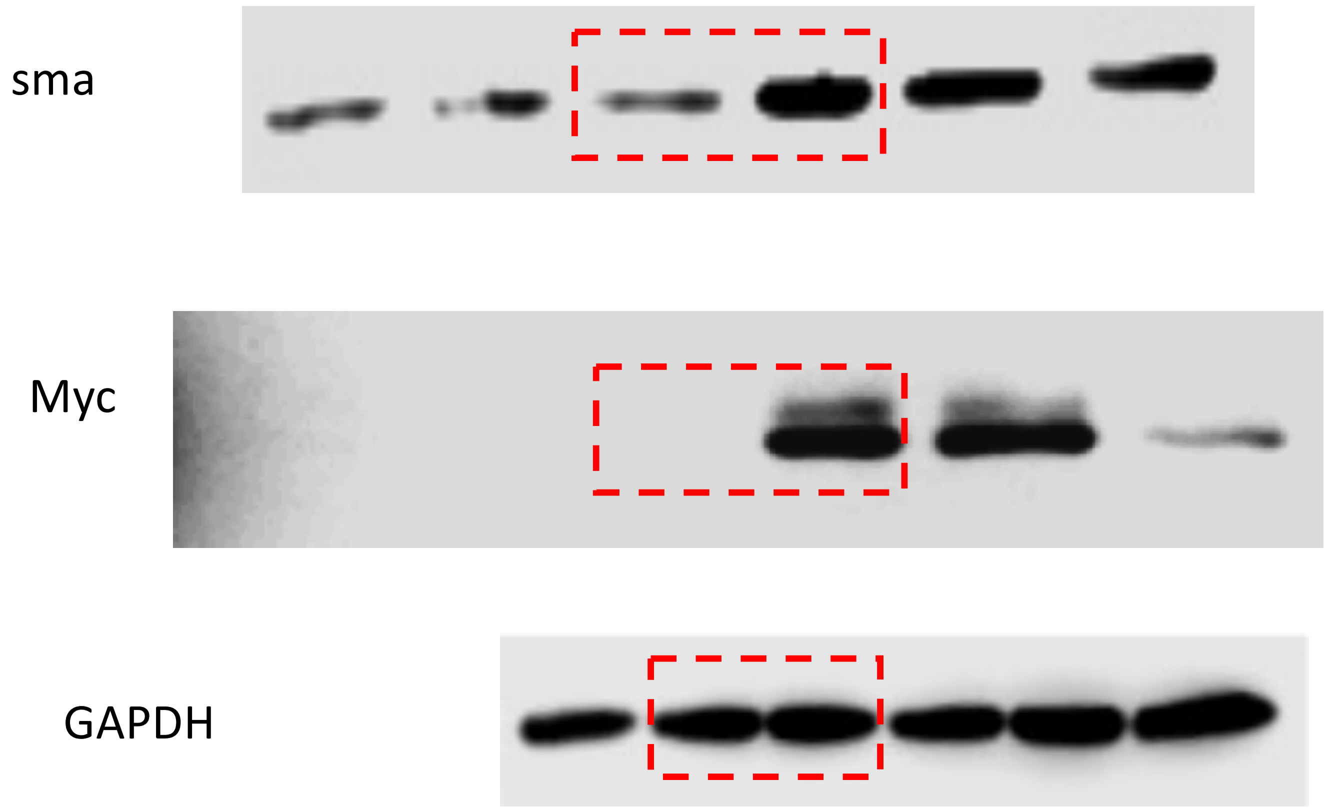

# Fig. 4a ACHN O/E – 72hrs

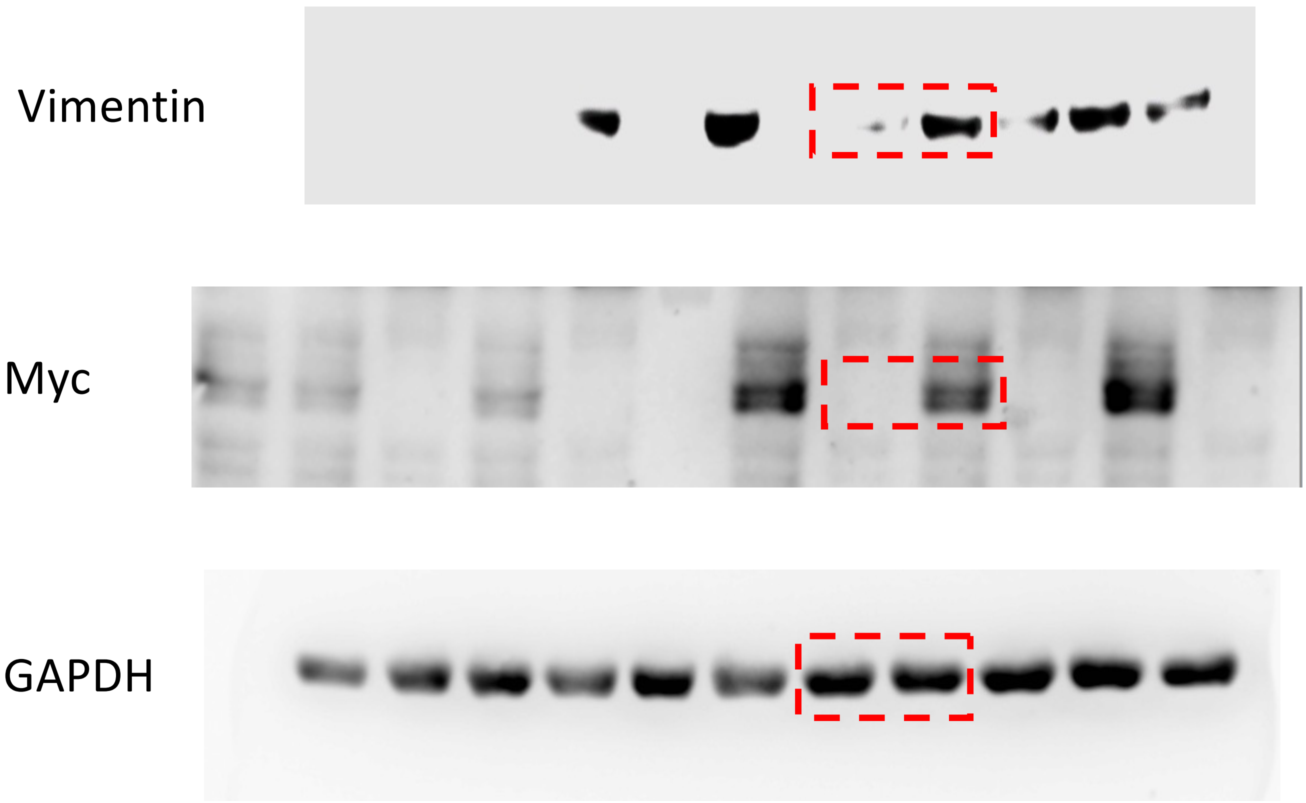

Fig. 4b 786-O O/E – 24hrs

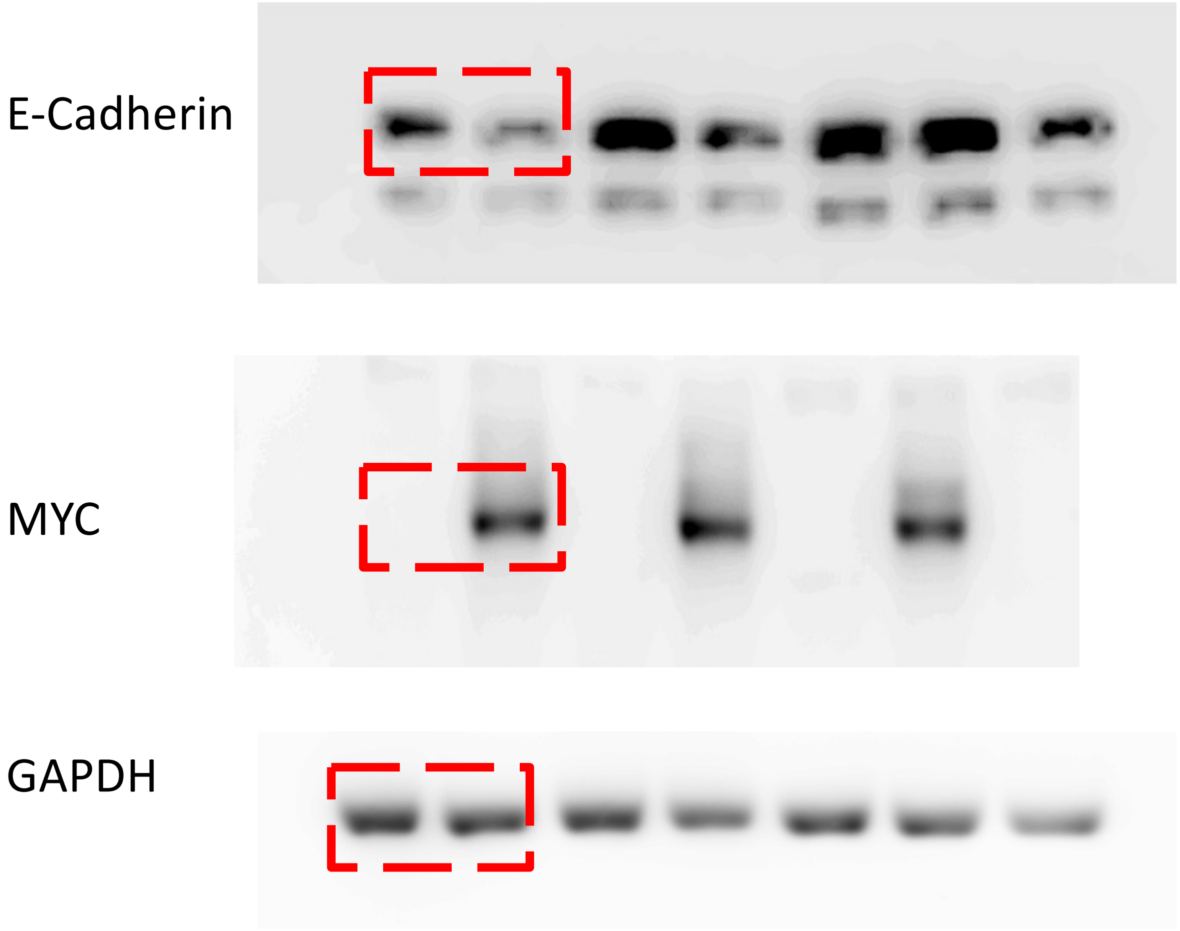

Fig. 4b 786-O O/E – 48hrs

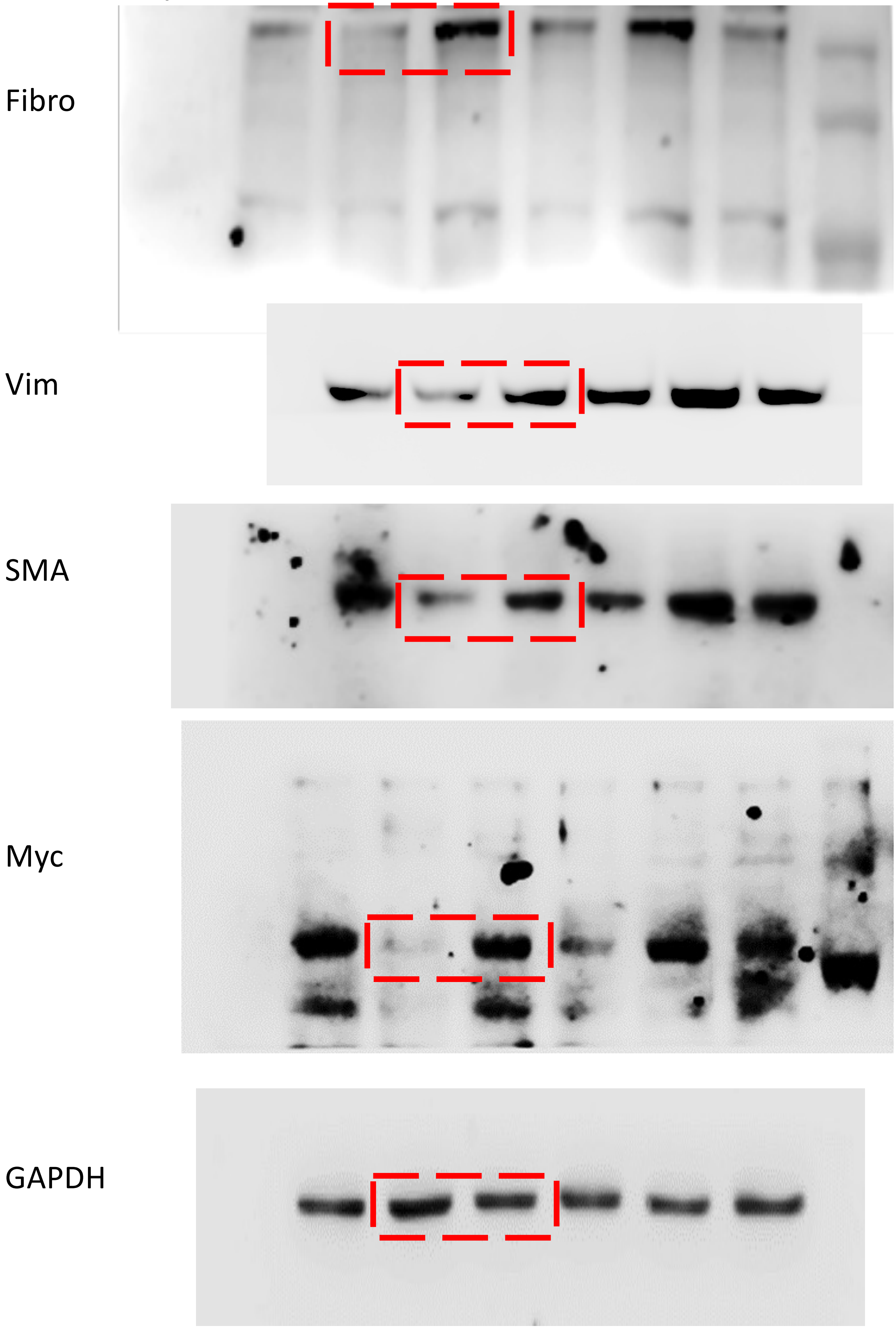

# Fig. 5a ACHN

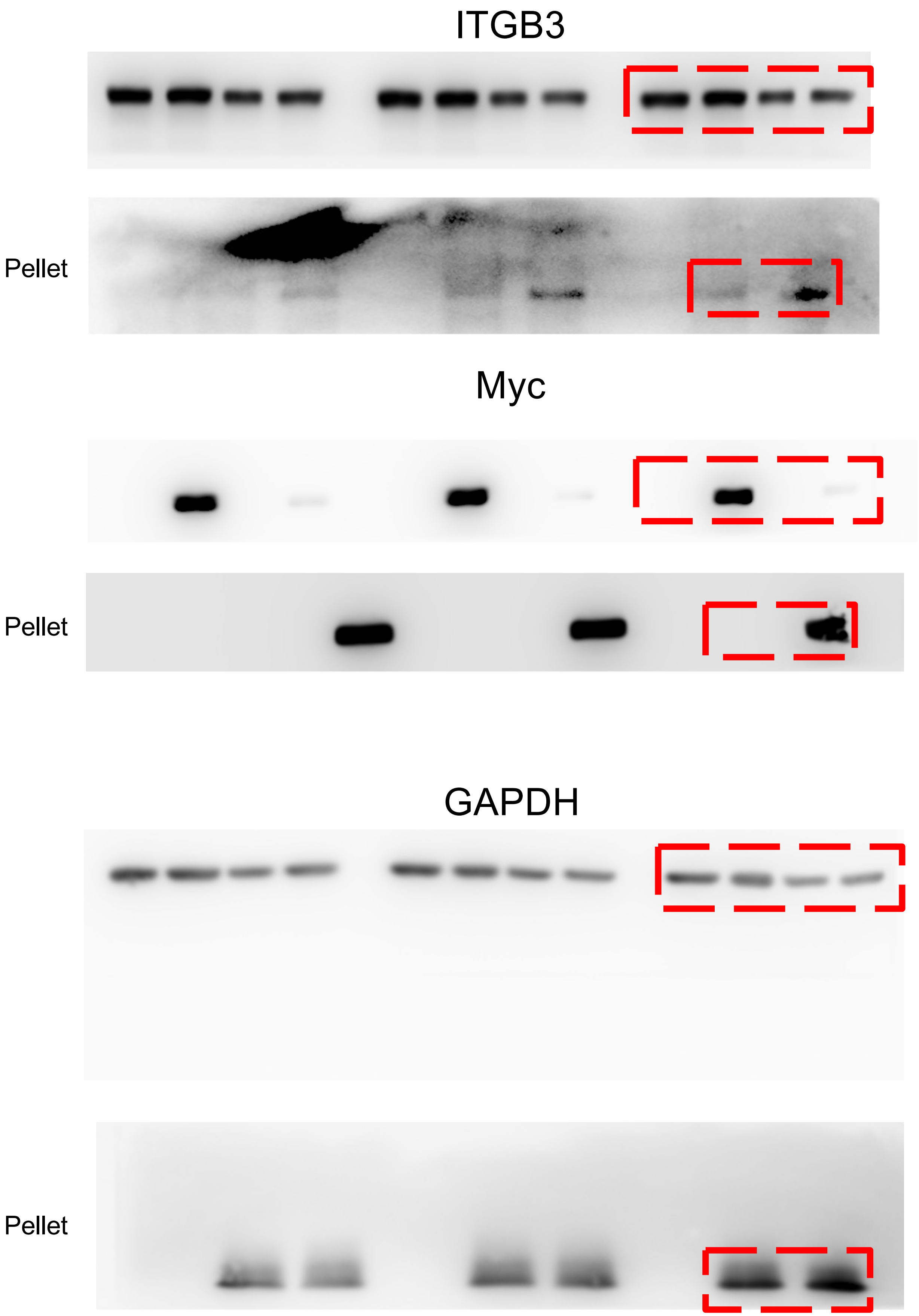

Fig. 5b 786-O

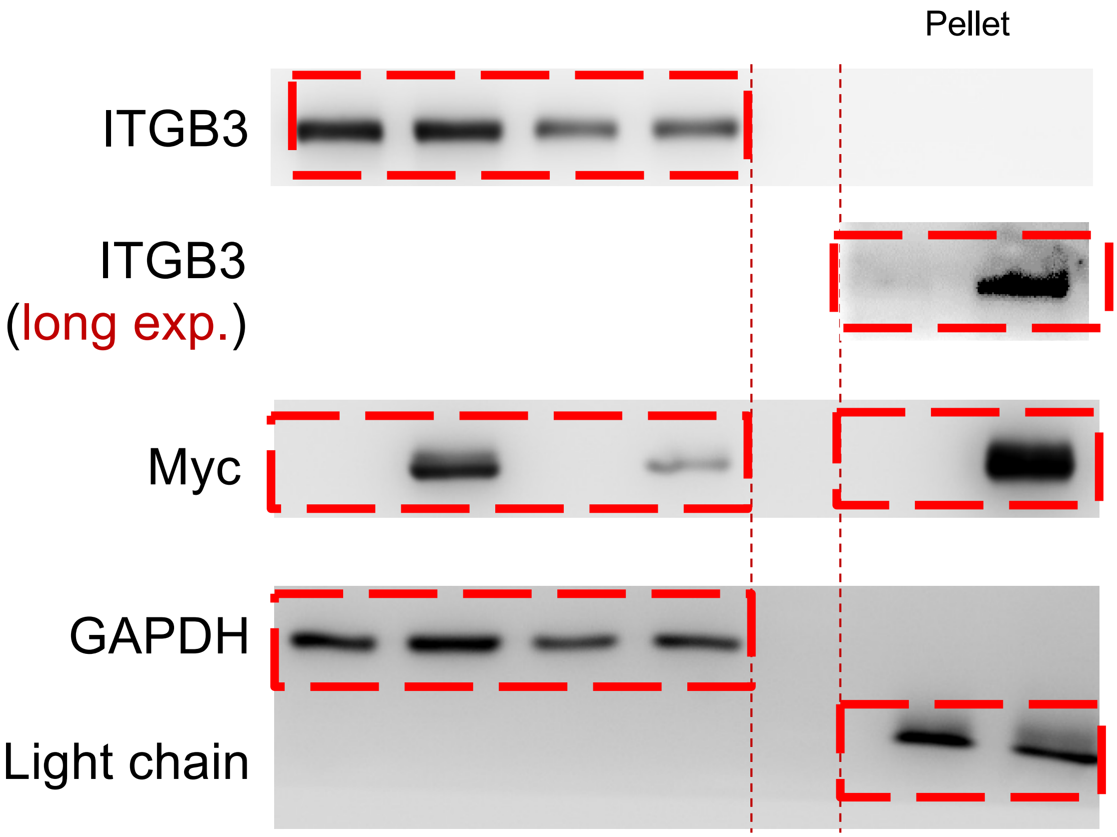

Fig. 6a ACHN

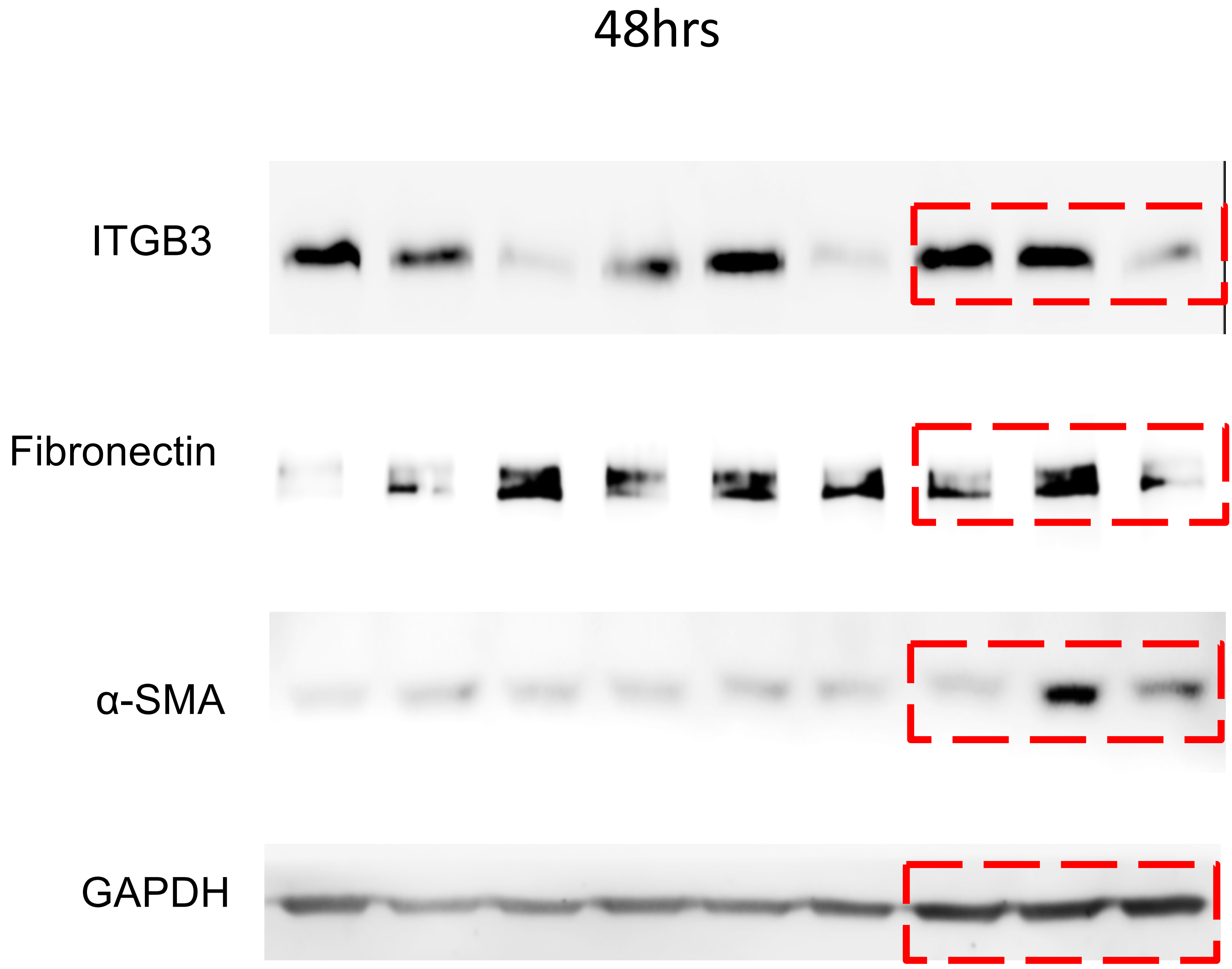

Fig. 6b 786-O

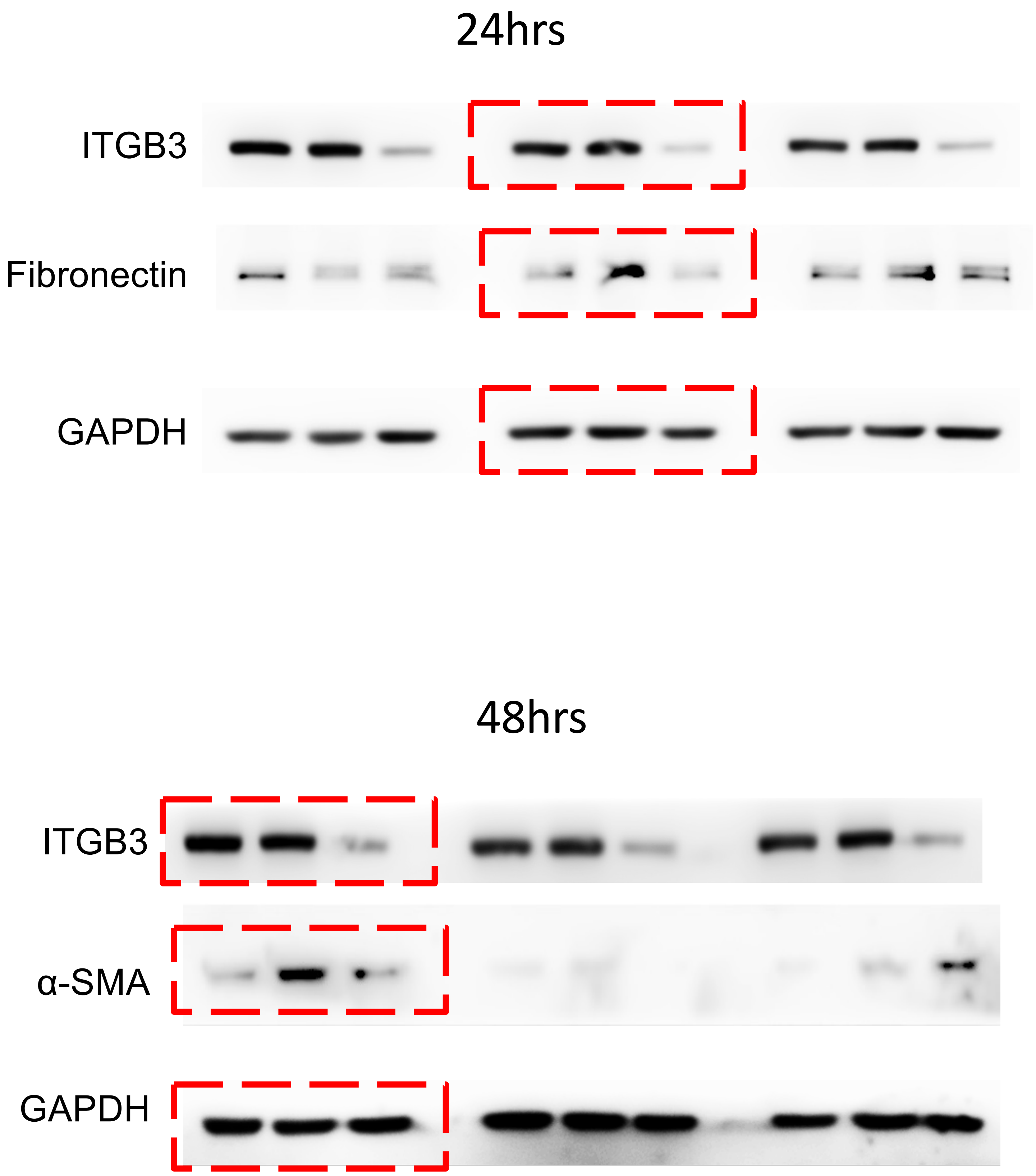

Fig. 7a, c ACHN

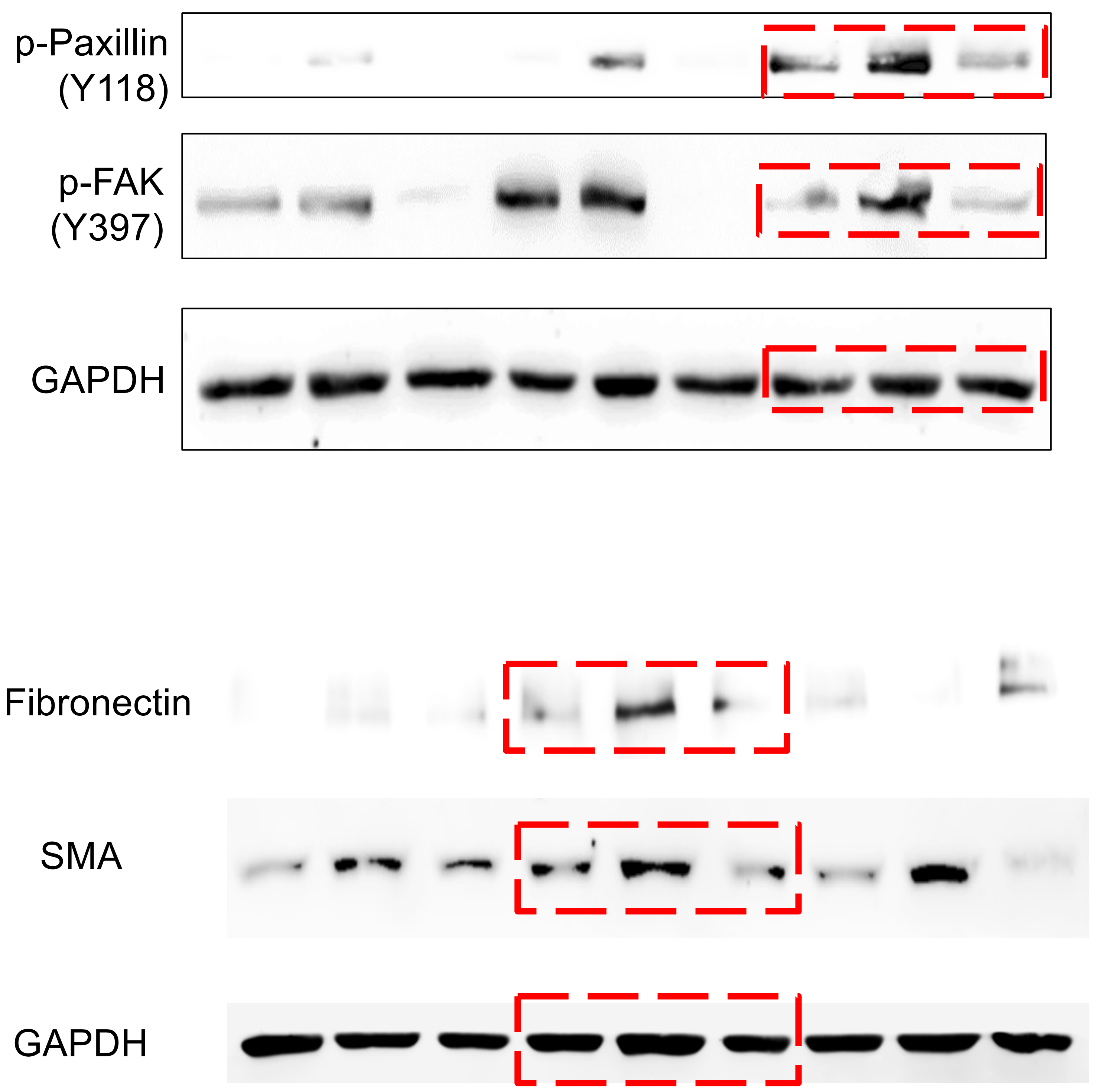

# Fig. 7b, d 786-O

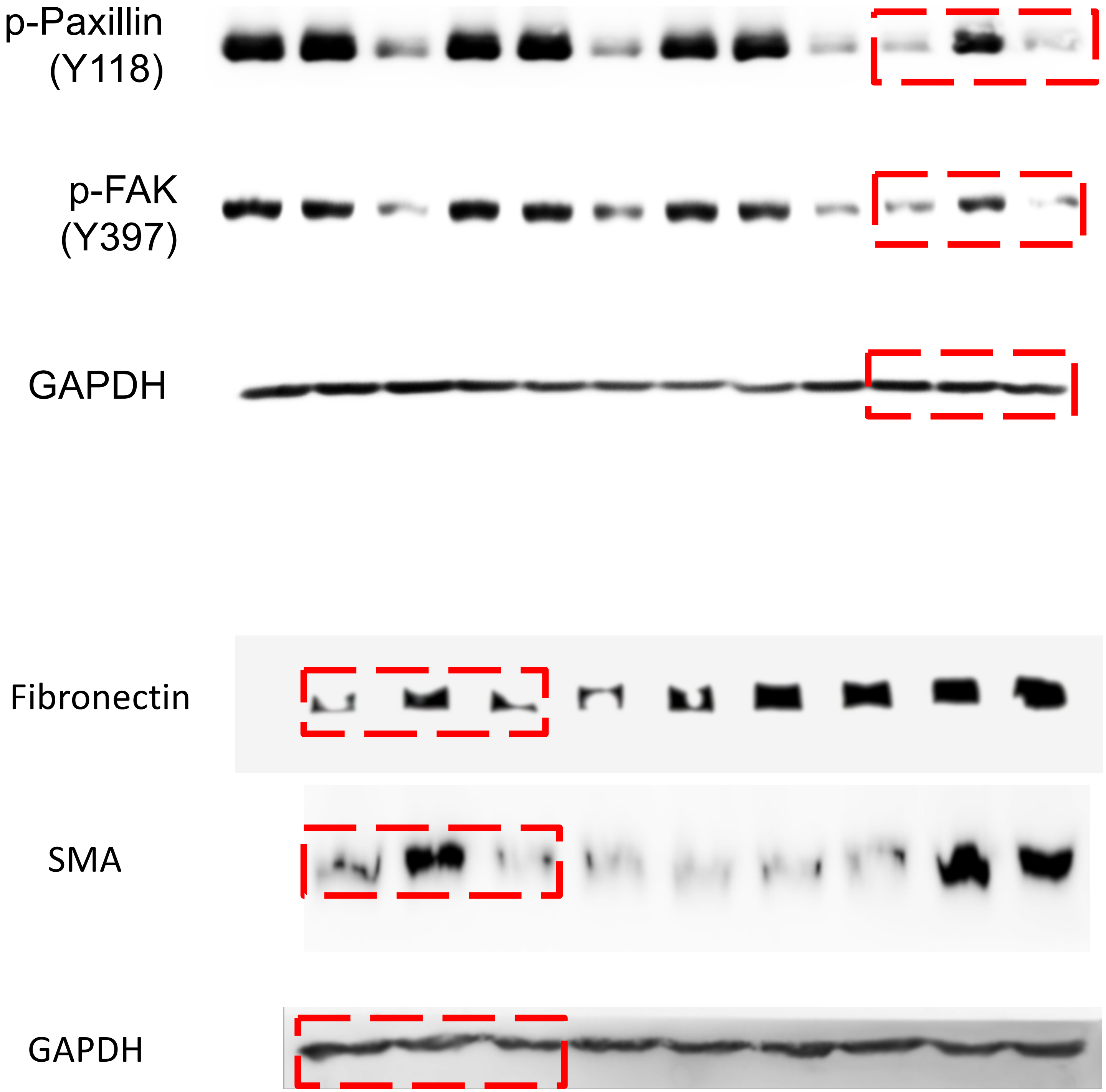

# Fig. 9a, c ACHN siRNA

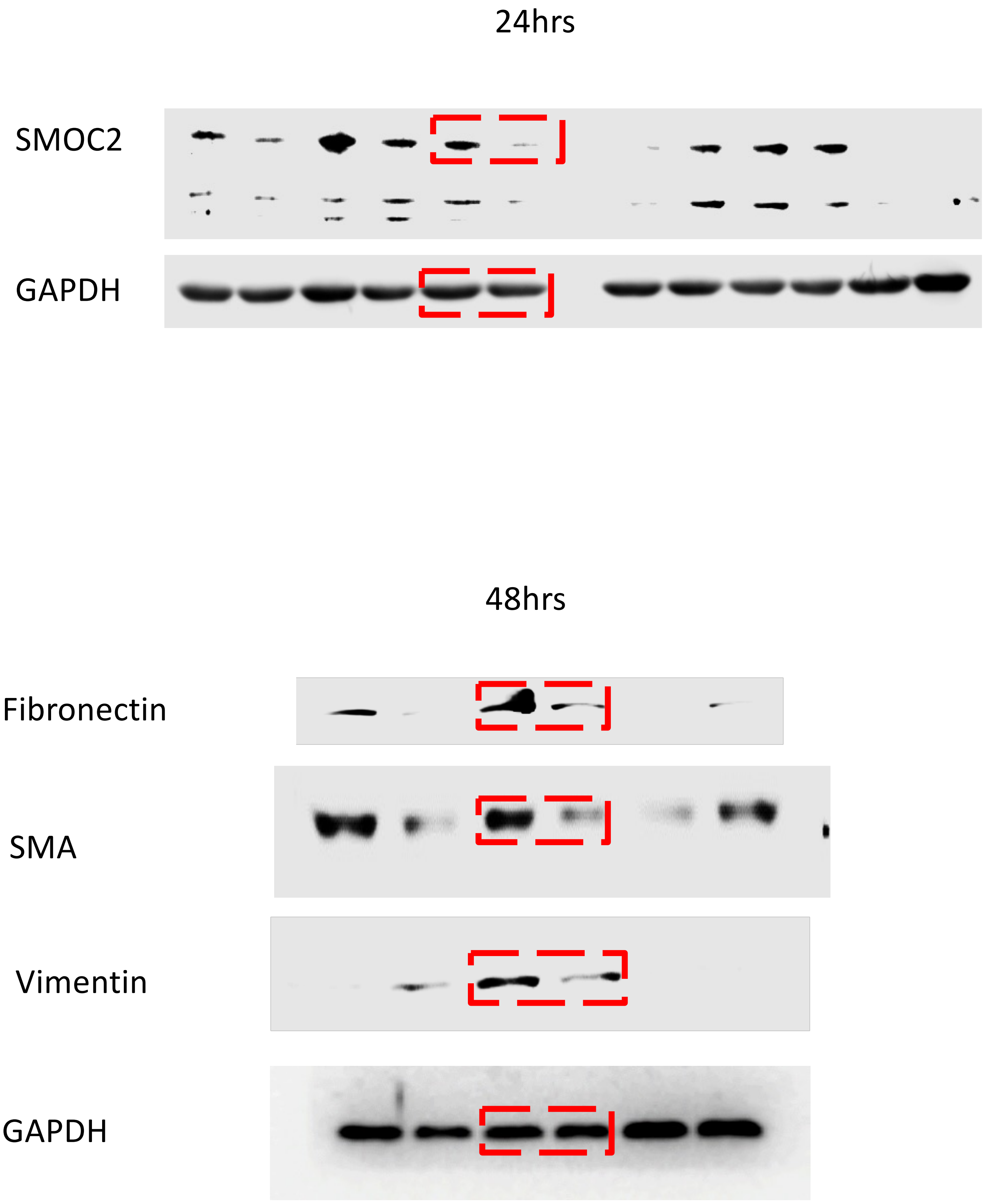

# Fig. 9b, d 786-O siRNA

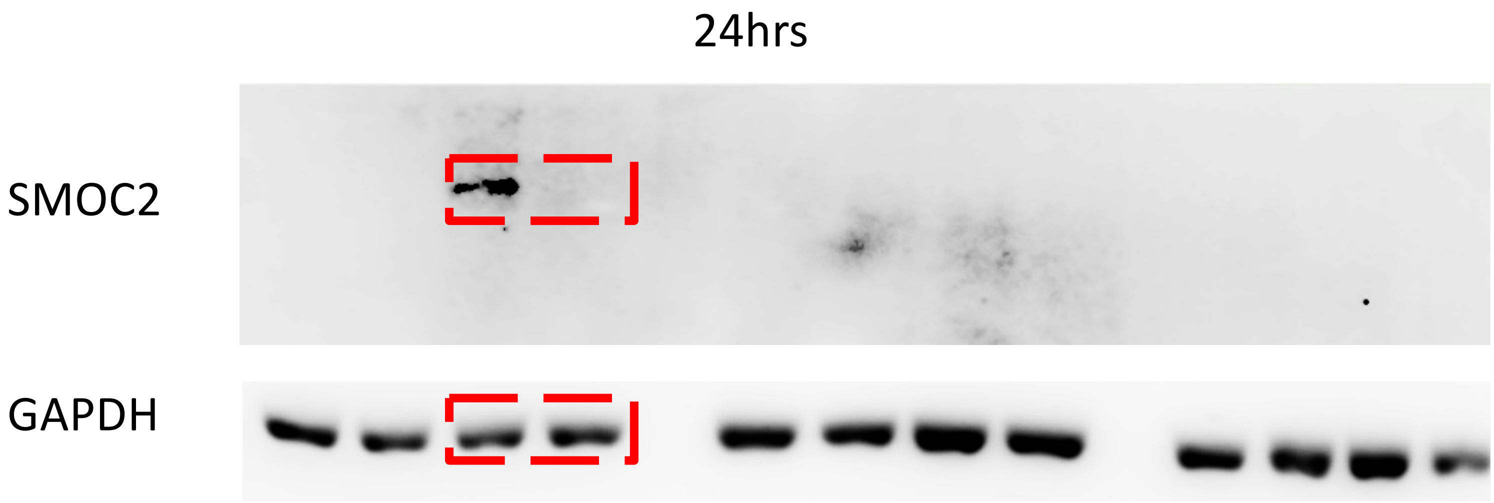


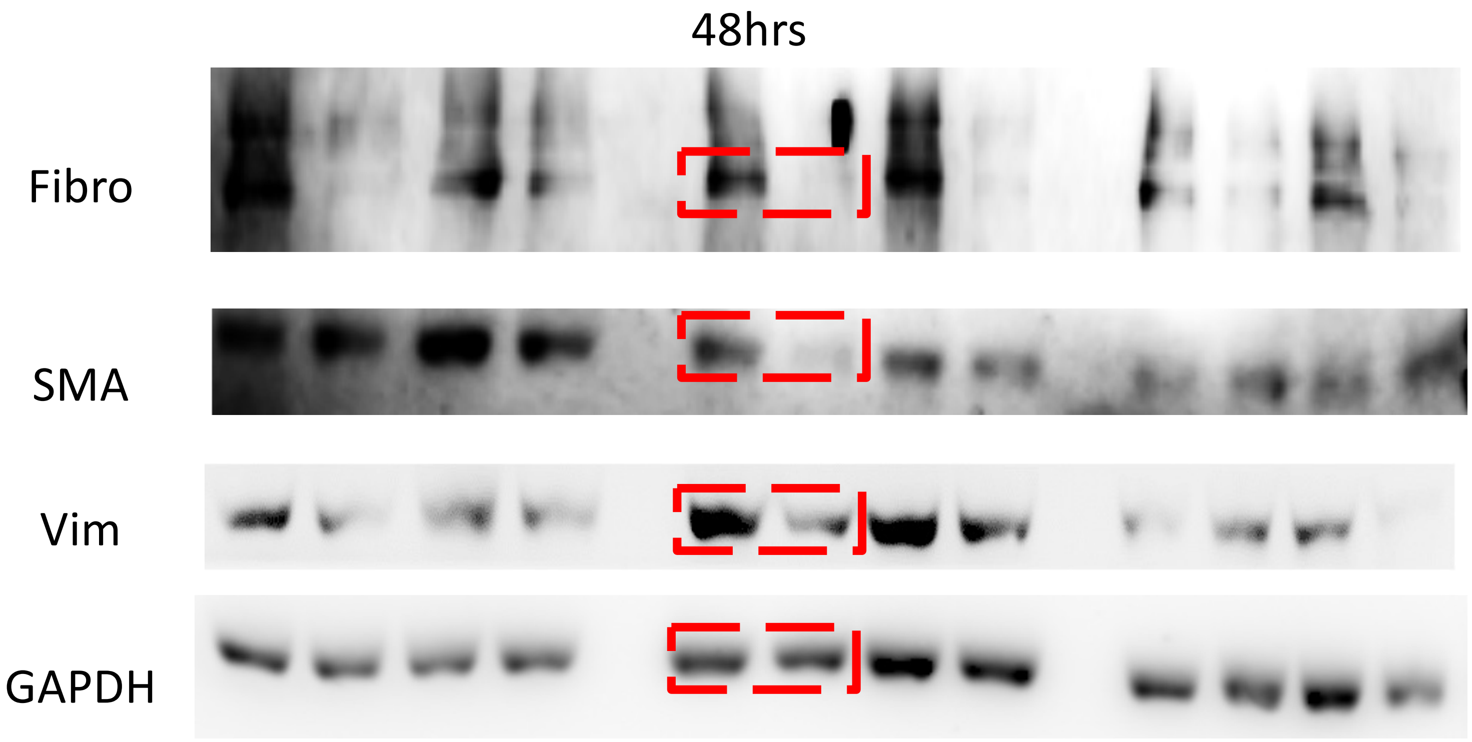

Supplement: Supplementary file 2 — Uncropped Western Blots [file 41419_2022_5059_MOESM2_ESM.pdf]
